# Protozoan‐Derived Cytokine‐Transgenic Macrophages Reverse Hepatic Fibrosis

**DOI:** 10.1002/advs.202308750

**Published:** 2024-01-21

**Authors:** Ying Chen, Jie Wang, Nan Zhou, Qi Fang, Haijian Cai, Zhuoran Du, Ran An, Deng Liu, Xuepeng Chen, Xinxin Wang, Fangmin Li, Qi Yan, Lijian Chen, Jian Du

**Affiliations:** ^1^ Department of Biochemistry and Molecular Biology Research Center for Infectious Diseases School of Basic Medical Sciences Anhui Medical University Hefei 230032 China; ^2^ The Provincial Key Laboratory of Zoonoses of High Institutions in Anhui Anhui Medical University Hefei 230032 China; ^3^ School of Nursing Anhui Medical University Hefei 230032 China; ^4^ Department of Anesthesiology The First Affiliated Hospital of Anhui Medical University Hefei 230032 China; ^5^ Department of Clinical Medicine Wannan Medical College Wuhu 241002 China; ^6^ GMU‐GIBH Joint School of Life Sciences The Guangdong‐Hong Kong‐Macau Joint Laboratory for Cell Fate Regulation and Diseases Guangzhou National Laboratory Guangzhou Medical University Guangzhou 510005 China

**Keywords:** immunotherapy, liver fibrosis, macrophages, polarization, *Toxoplasma gondii*, *Toxoplasma gondii* macrophage migration inhibitory factors

## Abstract

Macrophage therapy for liver fibrosis is on the cusp of meaningful clinical utility. Due to the heterogeneities of macrophages, it is urgent to develop safer macrophages with a more stable and defined phenotype for the treatment of liver fibrosis. Herein, a new macrophage‐based immunotherapy using macrophages stably expressing a pivotal cytokine from *Toxoplasma gondii*, a parasite that infects ≈ 2 billion people is developed. It is found that *Toxoplasma gondii* macrophage migration inhibitory factor‐transgenic macrophage (Mφ*^tgmif^*) shows stable fibrinolysis and strong chemotactic capacity. Mφ*^tgmif^* effectively ameliorates liver fibrosis and deactivates aHSCs by recruiting Ly6C^hi^ macrophages via paracrine CCL2 and polarizing them into the restorative Ly6C^lo^ macrophage through the secretion of CX3CL1. Remarkably, Mφ*^tgmif^* exhibits even higher chemotactic potential, lower grade of inflammation, and better therapeutic effects than LPS/IFN‐γ‐treated macrophages, making macrophage‐based immune therapy more efficient and safer. Mechanistically, *Tg*MIF promotes CCL2 expression by activating the ERK/HMGB1/NF‐κB pathway, and this event is associated with recruiting endogenous macrophages into the fibrosis liver. The findings do not merely identify viable immunotherapy for liver fibrosis but also suggest a therapeutic strategy based on the evolutionarily designed immunomodulator to treat human diseases by modifying the immune microenvironment.

## Graphical abstract

*Tg*MIF‐reprogrammed macrophages with stable pro‐resolution and strong chemotactic capacity effectively reverse liver fibrosis by modulating the immune microenvironment and promoting fibrinolysis. These findings not only identify viable immunotherapy for liver fibrosis but also suggest a therapeutic strategy based on the evolutionarily designed immunomodulator to treat human diseases.

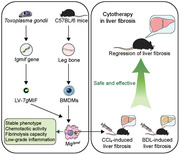

## 1 Introduction

Liver cirrhosis is a global problem that imposes a tremendous health and economic burden on many countries.^[^ ^1^ ^]^ Liver transplantation is the only curative therapy for cirrhosis, which is limited by the shortage of organ donors.^[^ ^2^ ^]^ Hepatic fibrosis is a crucial pre‐stage and reversible pathological process during the progression of cirrhosis.^[^ ^3^ ^]^ Early intervention is essential to stabilize disease progression.^[^ ^4^ ^]^ Although there is no standard and effective treatment for liver fibrosis under progress, various innovative therapeutic strategies for inhibiting and reversing hepatic fibrosis have been investigated and developed, including cell therapies.^[^ ^5^ ^]^ Macrophages play vital roles in orchestrating liver repair and regeneration and have been tested as a useful tool for immunotherapy in hepatic fibrosis.^[^ ^6^ ^]^ However, macrophages have the characteristics of plasticity and heterogeneity, and their phenotype and function may vary in response to the alteration of microenvironmental signals.^[^ ^7^ ^]^ Therefore, the potential risk of macrophage‐based immunotherapy is that the transplanted macrophages may undergo a phenotypic shift that is harmful in response to the microenvironment of the diseased organs.^[^ ^8^ ^]^ From this perspective, e*x vivo* polarized macrophages could have higher effectiveness and enhanced safety since their phenotypes are more uniform and appropriate. Notably, it has been shown that infusion lipopolysaccharides (LPS) and interferon‐γ (IFN‐γ) treated macrophages are more efficient than untreated macrophages or IL‐4‐induced macrophages in alleviating liver fibrosis. Mechanistically, LPS/IFN‐γ treated macrophages have a better ability to recruit host innate immune cells to relieve liver fibrosis synergistically.^[^ ^9^ ^]^ However, because LPS is the quintessential endotoxin and may induce systemic inflammation, its side effects and toxicities might restrict its clinical application. Therefore, it is urgent to develop safer macrophages with low inflammation and a high capacity to modify the immune microenvironment for the treatment of liver fibrosis, particularly in patients with local or systemic inflammation.

*Toxoplasma gondii* (*T. gondii*) is the most successful obligatory intracellular parasite that is capable of infecting nearly one‐third of the world's population.^[^ ^10^ ^]^ In order to widely spread in humans, it uses various strategies to survive, such as secreting immune‐associated molecules to modulate the host immune system. That is, *T. gondii* needs to employ various mechanisms to modulate the host immune system in order to prevent activation that may lead to their elimination and, at the same time, not cause serious immunosuppression that leads to the death of the host. The survival and transmission of parasites depend on their ability to evade or subvert the host immune system, including by modifying macrophage activities.^[^ ^11^ ^]^ *T. gondii*‐derived molecules are from evolutionary selection pressure formation in co‐evolution. Theoretically, these immune‐associated molecules secreted by *T. gondii* should be more stable, safer, and more effective to modulate human immunity.

Macrophage migration inhibitory factor (MIF) is a pluripotent cytokine of innate immunity. It has been identified to perform critical activities as an innate immune system mediator, contributing to inflammatory and immunological responses.^[^ ^12^ ^]^ Interestingly, some microbial pathogens also express a MIF‐like protein.^[^ ^13^ ^]^ Homologs of MIF have been discovered in many parasitic species, including *T. gondii*, and it has been suggested that parasites express MIF to manipulate the host immune response during infection.^[^ ^14^ ^]^ However, whether the homolog of MIF from *T. gondii* (*Tg*MIF) has the ability to regulate macrophage polarization and the underlying molecular mechanisms remain to be further explored.

This study aimed to explore a parasite‐derived immunomodulator as a potential immune cell programming agent for shaping macrophages. Then, we evaluated the safety and effectiveness of the novel immunotherapy in liver fibrosis.

## 2 Results

### 2.1 *Tg*MIF Promotes Macrophages With High Chemotactic and Fibrinolytic Potential

**Figure 1:**
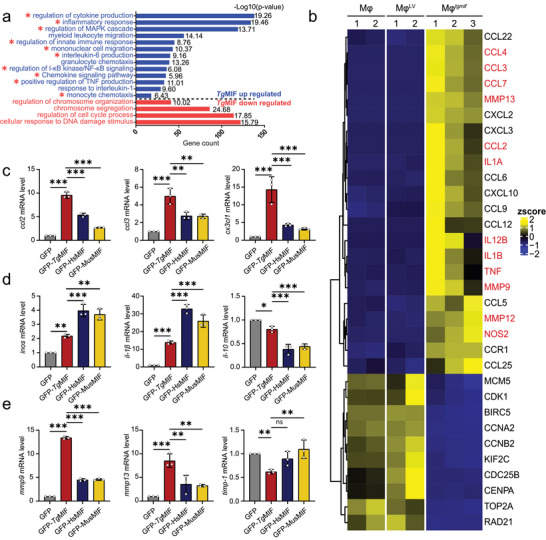
The phenotype of *Tg*MIF‐induced macrophages. a) Gene Ontology (GO) enrichment bar plot for differentially expressed genes between the Mφ^LV^ and Mφ*^tgmif^* groups. The pathways for genes upregulated in Mφ*^tgmif^* shown in blue, while the pathways for downregulated genes are shown in red. The ‐log10 (p value) was also displayed in each bar. b) Heatmap for representative differentially expressed genes across RAW264.7 (Mφ), Mφ*^tgmif^*, and Mφ^LV^ cells with biological replicates. The gene expression level was normalized by the z score. Color toward yellow indicates upregulation, and blue indicates downregulation. The significantly differentially expressed genes are marked in red. c–e) GFP, GFP‐*Tg*MIF, GFP‐*Mus*MIF, or GFP‐*Hs*MIF were transfected into RAW264.7 respectively. The relative expression levels of the indicated mRNAs were analyzed by qRT‐PCR (*n* = 3 per group). Results were analyzed using one‐way ANOVA. Bars = mean ± SD. *p < 0.05, **p < 0.01, ***p < 0.001 and ns, not statistically significant.

*Tg*MIF is a pluripotent cytokine that regulates the host immune response during *Toxoplasma* infection.^[^ ^14^ ^]^ As the first line of defense against *T. gondii*, macrophages are the most susceptible cells to the parasitic protozoan. Moreover, macrophages play an important role in the development of liver fibrosis. Therefore, we intended to investigate the effect of *Tg*MIF on macrophages. Using a lentiviral vector (LV) system, we successfully established a stably expressing *Tg*MIF macrophage cell line (Mφ*^tgmif^*) (Figure S1, Supporting Information). To further explore the phenotype of *Tg*MIF stably expressing macrophages, we used RNA‐Seq‐based transcriptome analysis to identify differentially expressed genes. We discovered that 1014 genes were upregulated and 388 genes were downregulated (Figure S2a, Supporting Information). According to gene ontology (GO) analysis, the differentially expressed genes were more enriched in the chemotactic signaling pathways, MAPK, and NF‐κB signaling pathways (**Figure** **1**a). The heatmap shows the differentially expressed genes of interest. *Tg*MIF promoted the expression of chemokines such as CCL3, CCL4, and CCL7; matrix metalloproteinases (MMPs) such as MMP9, MMP12, and MMP13, which promote fibrotic extracellular matrix (ECM) degradation; and some inflammatory cytokines such as IL1A (IL‐1α), IL1B (IL‐1β), NOS2 (iNOS), and TNF (TNF‐α) (Figure 1b). Then, we verified the key screened results on mouse bone marrow‐derived macrophages (BMDMs) and human monocyte‐derived macrophages (hMDMs). Consistent with the findings in RNA‐seq, expression of *Tg*MIF in both BMDMs (Figure S3a, Supporting Information) and hMDMs (Figure S3b, Supporting Information) also caused an increased expression of chemokines such as CCL3, CXCL2, and CCL2, as well as matrix metalloproteins like MMP9. Among these differentially expressed genes (DEGs), a fascinating observation focused our attention. The CCL2 expression level in Mφ*^tgmif^* was much higher (≈3691.5‐fold) than that in the control group (Figures 1b, S2b, Figure S3, Supporting Information). CCL2, also known as monocyte chemotactic factor 1 (MCP1), regulates the infiltration of monocyte‐derived macrophages (MoMFs) through combination with its specific receptor CCR2, which contributes to fibrosis dissolution in the repair phase.^[^ ^5^, ^15^ ^]^ The extremely high level of CCL2 in Mφ*^tgmif^* indicated that it might have the ability to modulate the microenvironment in vivo. According to the above results, *Tg*MIF appeared to promote macrophages with significant chemotactic and fibrinolytic potential and might produce inflammatory cytokines in vitro. Interspecies sequence alignments revealed that *Tg*MIF has only 26% identity with mammalian MIFs from host species,^[^ ^14^ ^]^ suggesting that *Tg*MIF's functions might be different from human MIF (*Hs*MIF). Therefore, we attempted to reprogram macrophages with either *Hs*MIF, mouse MIF (*Mus*MIF) or *Tg*MIF. However, in comparison to macrophages expressed *Tg*MIF, macrophages expressed *Hs*MIF and *Mus*MIF resulted in a lower level of chemokines such as *ccl2* and *ccl3* (Figure 1c), higher production of pro‐inflammatory cytokines (Figure 1d), lower expression of *mmps*, and a higher level of tissue inhibitor of metalloproteinase (TIMP)−1 (Figure 1e). Therefore, *Tg*MIF has a lower pro‐inflammatory potential and a higher chemotactic and fibrinolytic potential than *Hs*MIF and *Mus*MIF, implying that utilizing *Tg*MIF to reprogram macrophages has greater therapeutic potential than *Hs*MIF and *Mus*MIF.

### 2.2 Mφ*^tgmif^* Induces Strong Chemotactic Capacity and Low‐Grade Inflammation Compared with LPS/IFN‐γ‐treated Mφ

**Figure 2:**
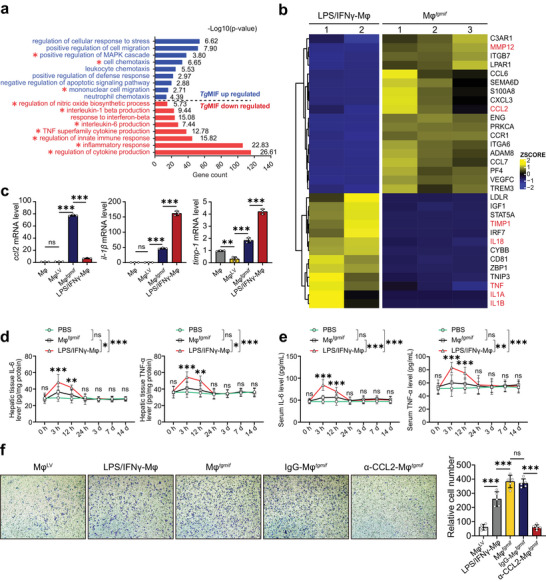
*Tg*MIF promotes macrophages with high chemotactic capacity and low‐grade inflammation compared with those activated by LPS/IFN‐γ. a) GO enrichment bar plot for differentially expressed genes between the Mφ*^tgmif^* and LPS/IFN‐γ‐Mφ groups. The pathways for genes upregulated in Mφ*^tgmif^* were shown in blue, and the downregulated pathways were shown in red. The ‐log10 (p value) was also displayed in each bar. b) Gene expression heatmap for representative differentially expressed genes across Mφ*^tgmif^* and LPS/IFN‐γ‐Mφ with biological replicates. The gene expression level was normalized by the z score. Color toward yellow indicates upregulation, and blue indicates downregulation. Significantly differentially expressed genes are marked in red. c) *ccl2, il‐1β*, and *timp‐1* mRNA expressions were analyzed by qRT‒PCR (*n* = 3 per group). d,e) Mice were infused with macrophages (2 × 10^6^ per mouse) and sacrificed humanely at the indicated time points after cell injection. PBS was injected as a control. The liver (d) and serum (e) were collected, and the concentrations of the indicated cytokines were determined by ELISA (*n* = 6 per group). f) C57BL/6 mice received 0.6 mL kg^−1^ body weight of CCl_4_ diluted in olive oil by i.p. injection twice per week to induce liver fibrosis. After the 8th CCl_4_ injection, PBMC were isolated and placed into the upper chamber; medium derived from different groups of macrophages was placed into the lower chamber for 24 h. CCL2‐neutralizing antibody (2 µg mL^−1^) or control IgG antibody (2 µg mL^−1^) was added into the medium of Mφ*^tgmif^* for 24 h (*n* = 5). Representative images and statistical analyses are shown (Magnification: 200 ×). Results were analyzed using one‐way ANOVA. Bars = mean ± SD. *p < 0.05, **p < 0.01, ***p < 0.001 and *ns*, not statistically significant.

A previous study showed that LPS/IFN‐γ treated macrophages are more efficient for cytotherapy in experimental liver fibrosis.^[^ ^9^ ^]^ However, these macrophages might produce a large amount of pro‐inflammatory cytokines in vivo, which have potential risks in clinical application. Endotoxin‐free *Tg*MIF‐transgenic macrophages may minimize the risk of endotoxin‐induced fever. To uncover the additional differences between LPS/IFN‐γ‐Mφ and Mφ*^tgmif^*, we performed RNA‐seq analysis and discovered that *Tg*MIF activated 615 genes and downregulated 489 genes compared to LPS/IFN‐γ‐Mφ (Figure S4, Supporting Information). *Tg*MIF‐upregulated genes were more enriched in the activation of the chemotactic pathway, according to GO analysis (**Figure** **2**a). Impressively, Mφ*^tgmif^* showed even higher levels of CCL2 expression than LPS/IFN‐γ‐Mφ (Figure 2b,c), indicating that Mφ*^tgmif^* may have higher chemotactic potential, assisting in the reversal of liver fibrosis. Besides, although Mφ*^tgmif^* did induce a slight increase in pro‐inflammatory factors such as TNF‐α, IL‐1β, and IL‐1α, these cytokines were considerably lower when compared to LPS/IFN‐γ‐Mφ (Figure 2b,c). This finding was consistent with the pro‐inflammatory cytokines in the liver tissue and serum of mice injected with macrophages through the tail vein. There was no significant difference in TNF‐α and IL‐6 levels between the PBS and Mφ*^tgmif^* groups. In contrast, pro‐inflammatory mediators both in liver tissue and serum significantly increased 3 h after LPS/IFN‐γ‐Mφ injection and decreased to baseline levels within 24 h (Figure 2d,e). Our results indicated that Mφ*^tgmif^* caused lower levels of inflammation than LPS/IFN‐γ‐Mφ both in vivo and in vitro. In addition, after 24 h, neither *Tg*MIF‐transgenic‐Mφ nor LPS/IFN‐γ‐Mφ induced the expression of pro‐inflammatory factors, and there was no statistical difference among the groups from 24 h to 14 days, suggesting that neither *Tg*MIF‐transgenic‐Mφ nor LPS/IFN‐γ‐Mφ could cause chronic inflammation. Further, the transwell chambers were used to determine the migratory ability of monocytes. Mouse peripheral blood mononuclear cells (PBMC) from fibrosis C57BL/6 mice were isolated and co‐cultured for 24 h with the medium derived from Mφ^LV^, Mφ*^tgmif^*, or LPS/IFN‐γ‐treated Mφ, respectively. The results suggested that the medium from Mφ*^tgmif^* strengthened the migration of PBMC compared to that from LPS/IFN‐γ‐treated Mφ and Mφ^LV^ (Figure 2f). In order to confirm whether CCL2 is a pivotal chemokine mediating Mφ*^tgmif^*‐induced PBMC migration, we added CCL2 neutralizing antibody or control IgG antibody into the medium. As expected, the results showed that CCL2 neutralization abolished the increased migration of PBMC by Mφ*^tgmif^* (Figure 2f). Together, these results demonstrated that Mφ*^tgmif^* had a stronger chemotactic capacity than LPS/IFN‐γ‐treated Mφ through the highly expressed CCL2. LPS/IFN‐γ‐treated Mφ caused a higher grade of inflammation in the short‐term than Mφ*^tgmif^*, yet neither could induce a long‐term inflammation.

### 2.3 Mφ*^tgmif^* Persists in the Liver for 7 Days and Attenuates Experimental Hepatic Fibrosis

**Figure 3:**
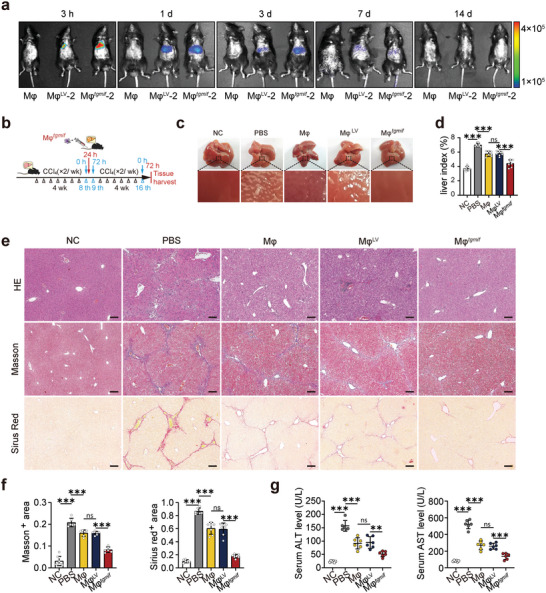
Mφ*^tgmif^* attenuates CCl_4_‐induced hepatic fibrosis in mice. a) Whole‐body imaging using in vivo imagin system at different time points. Mφ: fibrotic mice received RAW264.7; Mφ^LV^‐2: fibrotic mice received the Mφ^LV^‐2 stable cell line; Mφ*^tgmif^*‐2: fibrotic mice received the Mφ*^tgmif^*‐2 stable cell line. b. Study design: Mice were infused with 2 × 10^6^ macrophages (diluted in 150 µL PBS) or 150 µL PBS through the tail vein at 24 h post‐8th CCl_4_ injection. With continual injection with CCl_4_ for another 4 weeks, mice were sacrificed humanely 3 days after the last injection. c) Representative images of livers. d) The liver index was calculated according to the following formula: liver index (%) = liver weight (g)/body weight (g) × 100 (n = 7 per group). e) Representative histological liver sections with H&E, Masson's trichrome, and Sirius red staining. (×100; Scale bar = 200 µm). f) Positive areas were quantitatively analyzed (n = 6/6/7/7/7 (Sirius red) and n = 9/6/6/6/6 (Masson) per group). g) The serum concentrations of AST and ALT were determined by an automatic biochemical analyzer (*n* = 6 per group). Results were analyzed using one‐way ANOVA. Bars = mean ± SD. **p < 0.01, ***p < 0.001 and ns, statistically not significant. NC: negative control, mice were treated with an equal amount of pure olive oil; PBS: fibrotic mice receive PBS; Mφ: fibrotic mice received RAW264.7; Mφ^LV^: fibrotic mice received Mφ^LV^ stable cell line; Mφ*^tgmif^*: fibrotic mice received Mφ*^tgmif^* stable cell line.

To evaluate the effect of Mφ*^tgmif^* on liver fibrosis, we must ensure that the injected cells can enter the liver first. We employ less invasive delivery through the tail vein to minimize bleeding and vascular damage from hepatic artery or portal venous administration.^[^ ^16^ ^]^ Antares2‐expressing macrophages were established, which emitted intense, long‐wavelength luminescence suitable for in vivo monitoring (Figure S5a, Supporting Information). After being injected peripherally intravenously into hepatic fibrosis animals (Figure S5b, Supporting Information), the infused macrophages passed rapidly through the lungs in ≈10 minutes (Figure S5c, Supporting Information) and mostly accumulated in the liver ≈3 h after administration and persisted for 7 days (**Figure** **3**a). Immunofluorescent staining of the fresh‐frozen sections further confirmed the accumulation of infused macrophages in liver tissue 24 h after intravenous injection (Figure S5d, Supporting Information) and was barely detectable after 7 days of infusion (Figure S5e, Supporting Information). These results demonstrated that peripherally injected macrophages predominantly accumulated in the liver and survived long enough to exert a therapeutic effect. Next, the impact of Mφ*^tgmif^* on liver fibrosis was examined. Mice received different groups of macrophages at the mid‐stage of fibrogenesis. After another 4 weeks of CCl_4_ treatment, the mice were sacrificed humanely. Subsequently, blood and liver samples were harvested for further processing (Figure 3b). The liver images of Mφ*^tgmif^* ‐treated mice showed a smooth surface accompanied by a soft texture without granular substances (Figure 3c). Moreover, Mφ*^tgmif^* improved liver function indicators to almost normal levels, such as the liver index (Figure 3d), serum alanine aminotransferase (ALT), and aspartate transaminase (AST) (Figure 3g). H&E staining, Masson's trichrome staining, and Sirius red staining of liver sections showed that Mφ*^tgmif^* significantly reduced pathological lesions and collagen deposition. Despite the fact that other macrophage groups also showed decreased ECM deposition, which was consistent with published results,^[^ ^6^, ^9^ ^]^ Mφ*^tgmif^* exhibited the most effective function of alleviating liver fibrosis (Figure 3e,f). Collectively, these results revealed that peripherally injected Mφ*^tgmif^* predominantly accumulated in the liver and survived for 7 days to exert a therapeutic effect against hepatic fibrosis.

### 2.4 Mφ*^tgmif^* Promotes the Recruitment of Monocyte‐Derived Macrophages and a Phenotypic Switch to Ly6C^lo^ Macrophages to Induce Extracellular Matrix Degradation

**Figure 4:**
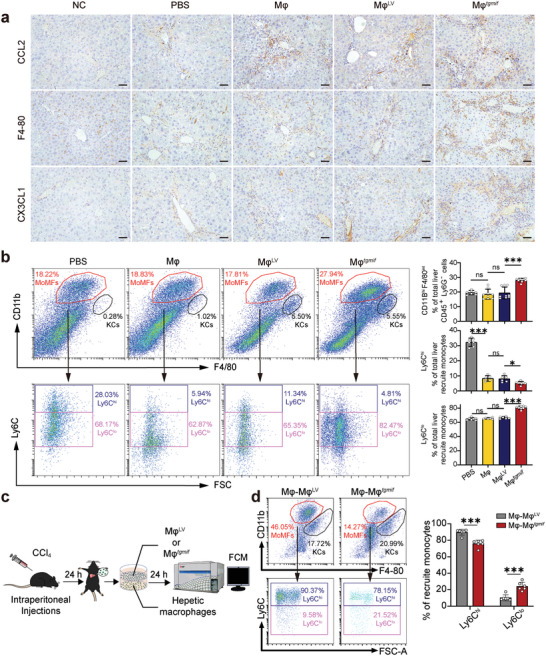
Mφ*^tgmif^* promotes the recruitment of MoMFs and a phenotypic switch to Ly6C^lo^ macrophages. a. The expression of CCL2, F4‐80, and CX3CL1 in livers tested by IHC (×200; Scale bar = 100 µm). b. Hepatic macrophages were isolated 24 h after cell infusion and analyzed by flow cytometry assay. Cells were gated to identify MoMFs (CD45^+^Ly6G^−^CD11b^hi^F4/80^int^) and KCs (CD45^+^Ly6G^−^CD11b^lo^F4/80^hi^). Representative flow cytometry density plots of KCs and MoMFs are shown. The corresponding statistical chart is on the right (n = 7/9/9/9 per group). These MoMFs were further divided into pro‐inflammatory MoMFs (Ly6C^hi^) and restorative MoMFs (Ly6C^lo^). Representative flow cytometry density plots of subsets (Ly6C^hi/lo^) are shown. The corresponding statistical chart is on the right (*n* = 7/6/6/8 per group). c) Following 24 h after CCl_4_ administration, hepatic macrophages were separated and co‐cultured with either Mφ*^tgmif^* or Mφ^LV^ for 24 h. d) Representative flow cytometry density plots of KCs, MoMFs, and subsets (Ly6C^hi/lo^) are shown. The corresponding statistical chart is on the right (*n* = 6 per group). Results were analyzed using one‐way ANOVA. Bars = mean ± SD. **p < 0.01, ***p < 0.001 and ns, not statistically significant. NC: negative control, mice were treated with an equal amount of pure olive oil; PBS: fibrotic mice received PBS; Mφ: fibrotic mice received RAW264.7; Mφ^LV^: fibrotic mice received the Mφ^LV^ stable cell line; Mφ*^tgmif^*: fibrotic mice received the Mφ*^tgmif^* stable cell line.

The recruitment of host innate immune cells is a critical mechanism for macrophage therapy.^[^ ^9^ ^]^ MoMFs are important mediators during fibrogenesis and are modulated by the chemokine receptor CCR2 and its ligand CCL2.^[^ ^15^, ^17^ ^]^ The detection of considerably elevated CCL2 expression in Mφ*^tgmif^* prompted us to explore the contribution of Mφ*^tgmif^* to MoMFs recruitment. In line with the in vitro findings, we found a substantial increase in CCL2 expression in Mφ*^tgmif^* ‐infused mice 24 h after cell injection compared to the other groups (**Figure** **4**a, Figure S6a,b, Supporting Information). Mφ*^tgmif^* delivery significantly increased F4/80^+^ macrophage infiltration, according to immunohistochemistry (IHC) staining (Figure 4a). Furthermore, a flow cytometry assay confirmed that Mφ*^tgmif^* administration enhanced the recruitment of MoMFs (CD45^+^Ly6G^−^CD11b^hi^F4/80^int^ macrophages) into the fibrotic liver (Figure 4b and Figure S6c, Supporting Information). Collectively, these results revealed that Mφ*^tgmif^* upregulated CCL2 expression both in vivo and in vitro, resulting in the enhanced recruitment of MoMFs to fibrotic livers. Infiltrating MoMFs are divided into two major subsets: Ly6C^hi^ macrophages, which are highly inflammatory and profibrotic macrophages, and Ly6C^lo^ macrophages, which are considered restorative macrophages that promote fibrotic degradation.^[^ ^17^, ^18^ ^]^ CX3CL1‐CX3CR1 axis exerts a key regulatory function in the transition from Ly6C^hi^ proinflammatory macrophages to Ly6C^lo^ alternative macrophages.^[^ ^19^ ^]^ We found that CX3CL1 was upregulated in Mφ*^tgmif^*‐treated mice (Figure 4a, Figure S6b, Supporting Information), and delivering Mφ*^tgmif^* not only boosted the recruitment of MoMFs but also dramatically increased the number of Ly6C^lo^ macrophages (Figure 4b). Then, we conducted an in vitro transition experiment to confirm this result. Following 24 h after CCl_4_ administration, hepatic macrophages were separated and co‐cultured with either Mφ*^tgmif^* or Mφ^LV^ for 24 h (Figure 4c). Results in vitro also indicated that Mφ*^tgmif^* could promote macrophage maturation and enhance the switch from Ly6C^hi^ macrophages to the Ly6C^lo^ subgroup (Figure 4d). Taken together, these results suggested that Mφ*^tgmif^* treatment resulted in the recruitment of MoMFs to the liver and promoted the functional switch from pro‐fibrogenesis to pro‐resolution by increasing CCL2 and CX3CL1 production. It is well known that Ly6C^lo^ macrophages represent the principle MMP‐expressing subset to promote matrix degradation.^[^ ^17b^ ^]^ Considering that *Tg*MIF elevated MMPs while downregulating TIMP‐1 in vitro (Figures 1b and 2b), we next decided to confirm these results in vivo (Figure S7a, Supporting Information). Consistently, the results showed that mice treated with Mφ*^tgmif^* had higher MMP2 and MMP9 expression but lower TIMP‐1 expression than the other groups (Figure S7b,c, Supporting Information). Our findings indicated that Mφ*^tgmif^* exerted antifibrosis effects not only through the direct role of injected macrophages but also through the modulation of the host immune microenvironment to increase the quantity of restorative macrophages, with the overall function of regulating the MMP/TIMP ratio to promote fibrinolysis.

### 2.5 Mφ*^tgmif^*, but not *Tg*MIF Itself, Deactivates Hepatic Stellate Cells (HSCs)

**Figure 5:**
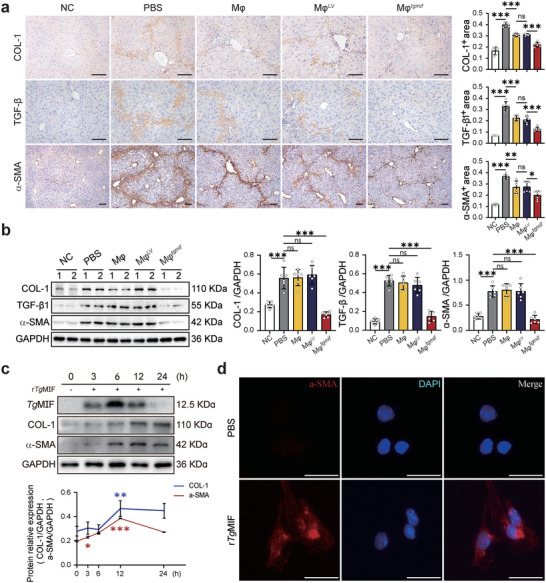
Mφ*^tgmif^*, but not r*Tg*MIF deactivates HSCs.a. Study design was described in Fig 3b. The liver tissue sections were stained with anti‐COL‐1, anti‐TGF‐β or anti‐α‐SMA antibodies for IHC (×200; Scale bar = 200 µm). Positively stained regions were quantitatively analyzed on the right (n = 6 per group (COL‐1); n = 5/6/6/6/6 (TGF‐β) and n = 6/6/6/6/7 (α‐SMA) per group). b. The relative protein expression in liver tissues was detected by Western blot (WB) analysis. Quantification was shown on the right side (n = 6). c. The indicated proteins were detected by WB analysis at 3 h, 6 h, 12 h, and 24 h after r*Tg*MIF (1 µg mL^−1^) was added to the culture medium of primary mouse HSCs. Quantification is shown in the lower panel (n = 3). d. The expression of α‐SMA in primary mouse HSCs was detected by immunofluorescence (IF) after 24 h of treatment with r*Tg*MIF (Scale bar = 25 µm). Results were analyzed using one‐way ANOVA. Bars = mean ± SD. *p < 0.05, **p < 0.01, ***p < 0.001 and ns, statistically not significant. NC: negative control, mice were treated with an equal amount of pure olive oil; PBS: fibrotic mice received PBS; Mφ: fibrotic mice received RAW264.7; Mφ^LV^: fibrotic mice received Mφ^LV^ stable cell line; Mφ^*tgmif*^: fibrotic mice received Mφ*^tgmif^* stable cell line.

Activated hepatic stellate cells (aHSCs) play pivotal roles in the onset and progression of liver fibrosis, which leads to increased ECM production. Hepatic fibrogenesis can be reduced by suppressing aHSCs or returning to a quiescent phenotype.^[^ ^20^ ^]^ To determine whether Mφ*^tgmif^* resolved liver fibrosis by influencing HSCs, liver sections were stained with aHSC markers, including alpha‐smooth muscle actin (α‐SMA), TGF‐β, and collagen I (COL‐1). The results showed that Mφ*^tgmif^* infusion significantly reduced the activation of HSCs compared to the other groups (**Figure** **5**a). This coincided with decreased mRNA and protein levels in the liver tissue of the Mφ*^tgmif^*‐treated group (Figure 5b and Figure S8a, Supporting Information). The TGF‐β level in peripheral blood also decreased in Mφ*^tgmif^* ‐treated mice (Figure S8b, Supporting Information). Considering that Mφ*^tgmif^* suppresses HSCs activation, it is unclear whether the *Tg*MIF protein deactivates HSCs independently of macrophages. Thus, we further detected the direct effect of *Tg*MIF on HSCs in vitro. Primary HSCs with a quiescent phenotype were isolated from normal C57BL/6 mice and treated with recombinant *Tg*MIF (r*Tg*MIF). r*Tg*MIF, as an exogenous protein, first appeared intracellularly at 3 h, peaked at 6 h, and then steadily reduced until it disappeared after 24 h. Surprisingly, we discovered that the r*Tg*MIF protein had the opposite effect on HSCs compared with Mφ*^tgmif^*. The expression of α‐SMA and COL‐1 increased in a time‐dependent manner after r*Tg*MIF protein treatment (Figure 5c). Similarly, immunofluorescent staining for α‐SMA also revealed that r*Tg*MIF appears to have the ability to activate HSCs (Figure 5d). *Tg*MIF overexpression in the human hepatic stellate cell line (LX‐2) also supported this finding (Figure S8c, Supporting Information). All of the above results demonstrated that Mφ*^tgmif^*, but not the *Tg*MIF protein itself, dismissed collagen deposition via deactivating aHSCs, indicating that Mφ*^tgmif^* is a preferable option for liver fibrosis therapy compared to r*Tg*MIF protein.

### 2.6 BMDMs*^tgmif^* Exhibit Better Safety Profiles and Therapeutic Effects than LPS/IFN‐γ Induced BMDMs in CCl_4_‐Induced Liver Fibrosis

**Figure 6:**
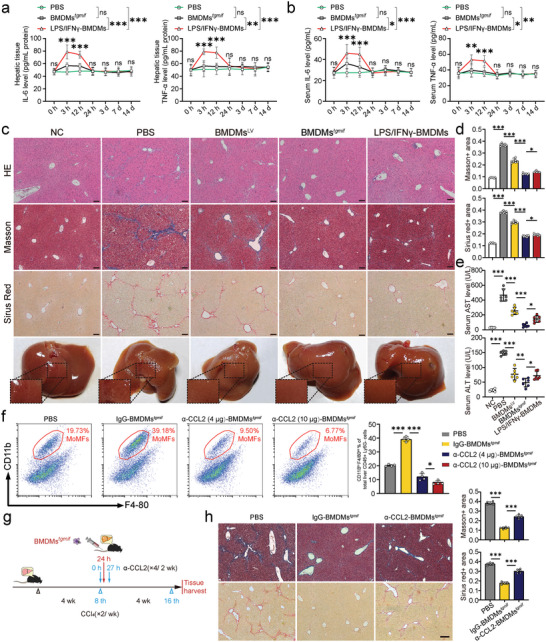
BMDMs^*tgmif*^ exhibits better safety profiles and therapeutic effects than LPS/IFN‐γ‐BMDMs in CCl_4_‐induced liver fibrosis. a,b. Mice were infused with BMDMs (2 × 10^6^ per mouse) and sacrificed humanely at the indicated time points after cell injection. PBS was injected as a control. The concentrations of the indicated cytokines in the liver (a) and serum (b) were determined by ELISA (n = 6 per group). c. Representative liver image, histological liver sections with H&E, Masson's trichrome, and Sirius red staining. (×100; Scale bar = 200 µm). Positive areas were quantitatively analyzed in d (*n* = 6 per group). e) The concentrations of biochemical indicators of liver function, including serum concentrations of ALT and AST (*n* = 6 per group). f) Representative flow cytometry density plots of MoMFs are shown following injection of CCL2 neutralizing antibody (4 µg or 10 µg per mouse, i.v.) 3 h after BMDMs*^tgmif^* delivery. The corresponding statistical chart is on the right (*n* = 4 per group). g) Anti‐CCL2 antibody (4 µg per mouse, i.v.) or an equal amount of control antibody (IgG2b) was given to the liver fibrosis mice 3 h after cell infusion, and this procedure was repeated twice a week for two weeks. h) Representative histological liver sections with Masson's trichrome, and Sirius red staining after CCL2 blockade (×100; Scale bar = 200 µm). Positive areas were quantitatively analyzed (*n* = 6 per group). Results were analyzed using one‐way ANOVA. Bars = mean ± SD. *p < 0.05, **p < 0.01, ***p < 0.001 and *ns*, statistically not significant. NC: negative control, mice were treated with an equal amount of pure olive oil; PBS: mice received PBS; BMDMs^LV^: mice received BMDMs overexpressing LV; BMDMs*^tgmif^*: mice received BMDMs overexpressing LV‐*Tg*MIF; LPS/IFN‐γ‐BMDMs: mice received BMDMs induced by LPS/IFN‐γ.

To facilitate clinical translation, we evaluated the safety and therapeutic effectiveness of BMDMs*^tgmif^* compared with LPS/IFN‐γ induced BMDMs. The experimental strategy for lentivirus‐transduced BMDMs and the expression of *Tg*MIF in groups of BMDMs was shown in Figure S9a,b (Supporting Information). To assess systemic safety, we measured inflammatory factors in the liver and serum for 14 days after the injection of macrophages (2 × 10^6^). The results showed that the mice receiving LPS/IFN‐γ‐BMDMs produced much higher levels of inflammatory cytokines, such as TNF‐α and IL‐6, both in the liver and serum than the mice receiving BMDMs*^tgmif^* (**Figure** **6**a,b), which matches the results in Figure 2d,e. Then, we treated mice with varying amounts and multiple transfers of BMDMs*^tgmif^* to test their systemic safety and toxicity. C57BL/6 mice were given three dosages of BMDMs (2 × 10^6^, 5 × 10^6^, and 7 × 10^6^ diluted in 150 µL PBS, respectively) or 150 µL PBS via the tail vein weekly for three weeks. Results showed that increasing the dosage and frequency of BMDMs*^tgmif^* injections had no effect on rectal temperature (Figure S10a, Supporting Information), body weight (Figure S10b, Supporting Information), or organ index (Figure S10c, Supporting Information), nor did it cause any microscopic tissue damage (Figure S10d, Supporting Information) or affect liver and kidney function (Figure S10e, Supporting Information), indicating that BMDMs*^tgmif^* has good biological security. Then, the therapeutic effects of different groups of BMDMs (2 × 10^6^) on liver fibrosis were evaluated according to the previous treatment plan (Figure 3b). The antifibrotic effect was assessed through serological tests (AST, ALT assays) and histochemical analysis (Figure 6c–e). The results indicated that both BMDMs*^tgmif^* and LPS/IFN‐γ‐BMDMs significantly ameliorated liver fibrosis, but BMDMs*^tgmif^* exhibited stronger therapeutic effects. As shown in Figure 4, Mφ*^tgmif^* upregulated CCL2 expression, resulting in the enhanced recruitment of MoMFs to fibrotic livers. In order to verify the role of CCL2 in the BMDMs*^tgmif^* treatment process, an in vivo CCL2 blocking experiment was conducted. Our results indicated that injection of CCL2 neutralizing antibody (4 µg or 10 µg per mouse, i.v.) significantly reduced the number of MOMFs entering into the liver 24 h after the injection of BMDMs*^tgmif^* (Figure 6f). Then, an anti‐CCL2 antibody (4 µg per mouse, i.v.) or an equal amount of control antibody (IgG2b) was given to the liver fibrosis mice 3 h after cell infusion, and this procedure was repeated twice a week for two weeks (Figure 6g). Selecting this time point was based on our data, which showed that the infused macrophages primarily accumulated in the liver for ≈3 h and were not detectable at 14 days post‐administration (Figure 3a). The results showed that CCL2 blockade significantly hindered the treatment effects of BMDMs*^tgmif^* on liver fibrosis (Figure 6h). Our findings demonstrated that the infusion of *Tg*MIF‐transgenic‐BMDMs mainly promoted the recruitment of MOMFs through the secretion of CCL2 to achieve the therapeutic effect.

### 2.7 BMDMs*^tgmif^* Exhibit Better Therapeutic Effects than LPS/IFN‐γ Induced BMDMs in BDL‐Induced Liver Fibrosis

**Figure 7:**
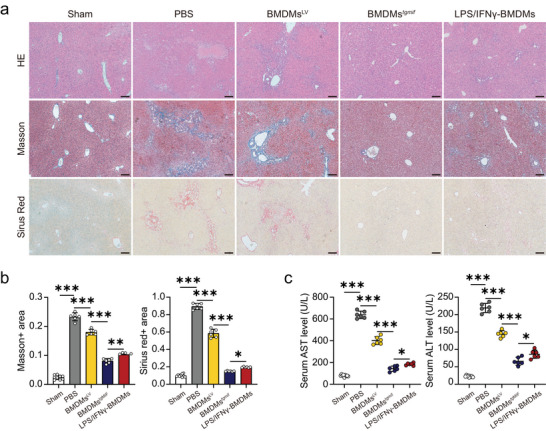
*Tg*MIF‐transgenic BMDMs alleviate BDL‐induced liver fibrosis. a) Representative histological liver sections with H&E, Masson's trichrome, and Sirius red staining. (×100; Scale bar = 200 µm). Positive areas were quantitatively analyzed in b) (*n* = 9/6/6/6/6 per group). c) The concentrations of biochemical indicators of liver function, including serum concentrations of ALT and AST (*n* = 6 per group). Results were analyzed using one‐way ANOVA. Bars = mean ± SD. *p < 0.05, **p < 0.01, ***p < 0.001 and *ns*, statistically not significant. Sham: sham‐operated control mice; PBS: mice received PBS; BMDMs^LV^: mice received BMDMs overexpressing LV; BMDMs*^tgmif^*: mice received BMDMs overexpressing LV‐*Tg*MIF; LPS/IFN‐γ‐BMDMs: mice received BMDMs induced by LPS/IFN‐γ.

The cholestatic liver fibrosis model was prepared by subjecting C57BL/6 mice to bile duct ligation (BDL). BMDMs were infused via the tail vein of the mice 10 days after the BDL operation. The mice were sacrificed humanely 11 days after the cell infusion for further analysis. Compared to sham mice, BDL mice showed severe changes in liver morphology, including necrosis of liver cells, severe bile duct hyperplasia, portal edema, and marked fibrosis (**Figure** **7**a,b). The levels of ALT and AST in the serum of BDL mice were much higher than those of sham mice (Figure 7c). These changes in liver morphology and liver enzymes were markedly improved by *Tg*MIF‐transgenic BMDMs treatment (Figure 7). Collectively, our findings showed that BMDMs*^tgmif^* displayed higher therapeutic effectiveness than LPS/IFN‐γ induced BMDMs.

### 2.8 *Tg*MIF Promotes CCL2 Expression By Activating the ERK/HMGB1/NF‐κB Pathway

**Figure 8:**
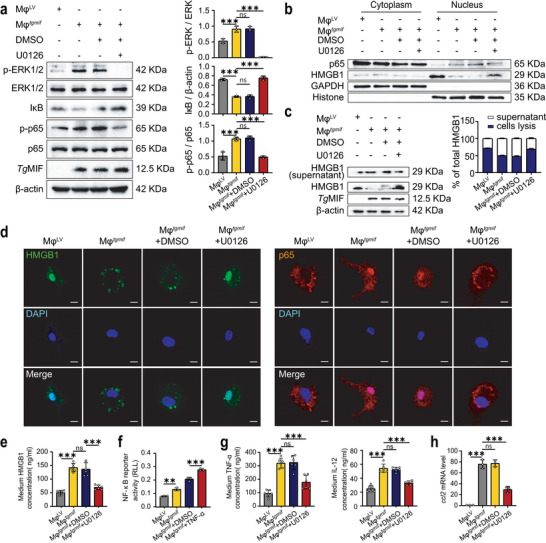
*Tg*MIF upregulates the expression of CCL2 through the ERK/HMGB1/NF‐κB pathway. a) WB analysis was used to determine the expression of proteins related to the ERK and NF‐κB pathways. Quantification was shown on the right side (*n* = 3 per group). b) Nuclear and cytoplasmic separation experiments showed the nuclear‐cytoplasmic shift of HMGB1 and p65. c) WB analysis was utilized to detect the level of HMGB1 in the whole cell lysate and cell culture supernatant. Quantification was shown on the right side (*n* = 3 per group). d) IF showed the nuclear and cytoplasmic translocation of HMGB1 and p65 (Scale bar = 10 µm). e) ELISA detected the secretion of HMGB1 in the supernatant of cell culture (*n* = 6 per group). f). NF‐κB activation was determined using a luciferase reporter assay (*n* = 3 per group). g) ELISA was used to detect the secretion of inflammatory factors in the supernatant of cell culture (*n* = 6 per group). h) ccl2 mRNA expressions were analyzed by qRT‒PCR (*n* = 3 per group). Results were analyzed using one‐way ANOVA. Bars = mean ± SD. **p < 0.01, ***p < 0.001 and *ns*, not statistically significant.

**Figure 9:**
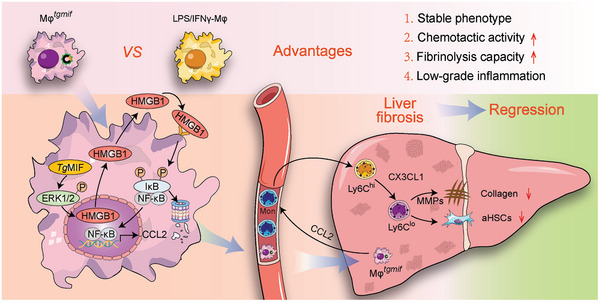
Mφ*^tgmif^* with strong chemotactic capacity and lower grade of inflammation on the usage of liver fibrosis. By activating the ERK/HMGB1/NF‐κB pathway, *Tg*MIF promoted CCL2 expression, which enabled Mφ*^tgmif^* to recruit Ly6C^hi^ macrophages into the liver. Subsequently, Mφ*^tgmif^* polarized Ly6C^hi^ into the restorative Ly6C^lo^ macrophage through the secretion of CX3CL1, with the overall function of efficiently alleviating liver fibrosis and deactivating aHSCs.

Given that we have screened out MAPK and NF‐κB pathways induced by *Tg*MIF using RNA‐seq, we next aimed to uncover the molecular mechanism underlying the high expression of CCL2. CCL2 is a well‐established target chemokine of the transcription factor NF‐κB.^[^ ^21^ ^]^ ERK pathway is the main signaling cascade among MAPK signal pathways.^[^ ^22^ ^]^ Here, we demonstrated that *Tg*MIF promoted the phosphorylation of ERK, led to the phosphorylation of the NF‐κB p65 subunit, and degradation of IκB occurred, indicating that *Tg*MIF activates NF‐κB. In addition, U0126, an ERK inhibitor, significantly inhibited ERK phosphorylation, p65 phosphorylation, and IκB degradation in *Tg*MIF stably expressing macrophages, indicating that *Tg*MIF triggered the phosphorylation of ERK and the subsequent NF‐κB pathway (**Figure** **8**a). HMGB1 has been shown in many studies to induce inflammation through the MEK/ERK signaling pathway,^[^ ^23^ ^]^ and be a critical component in the HMGB1/TLR4/NF‐κB axis.^[^ ^23^, ^24^ ^]^ Therefore, we attempted to investigate the role of HMGB1 in the *Tg*MIF‐induced NF‐κB pathway. The stable expression of *Tg*MIF increased the translocation of HMGB1 from the nucleus to the cytoplasm and eventual extracellular secretion (Figure 8b–e). Consistently, analyses of cytoplasmic and nuclear p65 levels revealed that p65 translocation into the nucleus was concomitant with the release of HMGB1 in *Tg*MIF‐expressing cells (Figure 8b,d). U0126 treatment reduced ERK phosphorylation and attenuated HMGB1 release and then inhibited subsequent NF‐κB activation in Mφ*^tgmif^* (Figure 8b–e,g). Inhibiting ERK signaling in *Tg*MIF‐transgenic‐Mφ also decreased CCL2 expression (Figure 8h). All these results demonstrated that *Tg*MIF phosphorylated ERK, which facilitates HMGB1 release from the nucleus to the extracellular matrix, leading to the degradation of IκB and translocation of p65 into the nucleus to activate the NF‐κB pathway, which contributed to the highly expressed CCL2 (**Figure** **9**).

## 3 Discussion

*T. gondii* is one of the most widespread parasitic protozoans, infecting almost all warm‐blooded animals and nearly one‐third of the world's population. *T. gondii* infection is usually asymptomatic in immunocompetent adults but can cause lethal toxoplasmosis in immunocompromised individuals.^[^ ^10^, ^11^ ^]^ The survival of the host not only facilitates the long‐term survival of parasite encystation but also provides a niche for the parasite to persist until the opportunity for transmission to other hosts arises.^[^ ^10^, ^11^ ^]^ According to evolutionary optimization theory, the marginal benefit of evolving increased parasite resistance must be balanced against the marginal costs imposed by the immune response.^[^ ^25^ ^]^ Hypothetically, to balance advantages and costs, humans may have acquired a specific immune response against *T. gondii*. Meanwhile, *T. gondii* may have evolved a protective mechanism against fibrosis, preserving the host and delaying the elimination of parasites. The pathogen *T. gondii* co‐opts host immunity by secreting a variety of effector proteins into host cells, one of which is *Tg*MIF. Our results demonstrated for the first time that *Tg*MIF affected the host immune response by phosphorylating ERK and promoting HMGB1 release from the nucleus to the extracellular matrix, leading to degradation of IκB and release of p65 into the nucleus to activate the NF‐κB pathway. The NF‐κB signaling pathway is a critical regulator of the host's innate immune system. NF‐κB activation may suppress apoptosis and enhance cell migration, allowing for cell proliferation for further infection.^[^ ^26^ ^]^ The *Tg*MIF‐induced pro‐inflammatory response may not only reduce parasite burdens in the host but also cause tissue damage, allowing pathogens to bypass tissue barriers and disseminate to other host tissues, which may explain *T. gondii*’s symbiotic relationship with humans.

Some studies have recently attempted to utilize parasites or their derivatives to treat human diseases.^[^ ^27^ ^]^ ES‐62, a molecule secreted by filarial nematodes, is able to protect mice from mast cell‐dependent hypersensitivity in the skin and lungs.^[^ ^28^ ^]^ Anti‐inflammatory protein‐2 (AIP‐2), secreted by hookworms, suppressed airway inflammation in a mouse model of asthma.^[^ ^29^ ^]^ In preclinical melanoma mouse models, treatment with *T. gondii* GRA17 knockout tachyzoites and anti‐PD‐L1 therapy together significantly suppressed tumor growth and extended mouse survival.^[^ ^30^ ^]^ However, utilizing live parasites to treat human illnesses has potential risks, as replicated pathogens cannot be removed by immunocompromised hosts. By this means, it is worthwhile to investigate some parasite‐derived immunomodulators as potential immune cell programming agents for treating human diseases. Most significantly, this strategy separates benefits from damage and eliminates the need to introduce a potentially pathogenic parasite in the treatment of disease.^[^ ^31^ ^]^

Cell therapy has been explored as an alternative therapeutic strategy to increase the survival of patients with liver disease.^[^ ^18b^ ^]^ Transfusion of macrophages showed therapeutic effects of relieving fibrogenesis,^[^ ^6b‐f^ ^]^ Translational studies have already demonstrated that primary human MoMFs sourced from healthy donors have antifibrotic activity after cell transfer to fibrotic immunocompromised mice.^[^ ^6a^ ^]^ Macrophages are highly plastic, and their functional phenotypes change in a microenvironment‐dependent manner. Therefore, infusion of polarized macrophages may have a better therapeutic effect. Previous research has demonstrated that LPS/IFN‐γ‐treated macrophages are more effective in hepatic fibrosis therapy than untreated or IL‐4‐treated macrophages,^[^ ^9^ ^]^ indicating that more defined macrophages result in more effective therapy. Nevertheless, this treatment could have safety concerns in cirrhotic patients with local or systemic inflammation. In this study, we identified *Tg*MIF as a novel and effective parasite‐derived immunomodulator to design macrophages, which have better therapeutic potential than LPS/IFN‐γ‐treated macrophages or the macrophage population as a whole. For one thing, the stable expression of *Tg*MIF in macrophages can promise therapeutic effects that last for a longer period. For another, Mφ*^tgmif^* shaped with an antigenic molecule derived from microorganisms in nature, exhibits some special advantages, such as stronger chemotactic potential, over LPS/IFN‐γ‐Mφ. More importantly, Mφ*^tgmif^* is safer than LPS/IFN‐γ‐Mφ because it induces much lower secretion of pro‐inflammatory cytokines like TNF‐α and IL‐6 in vivo compared with BMDMs induced by LPS/IFN‐γ, suggesting that they may be more appropriate for treating cirrhotic patients with local or systemic inflammation. Therefore, Mφ*^tgmif^* infusion appears to be safer and more effective for anti‐fibrotic treatment.

Previous studies and our results confirmed that infused macrophages predominantly accumulated in the liver.^[^ ^6^, ^32^ ^]^ However, these cells injected peripherally are not enough to exert such a long‐lasting antifibrosis effect. An important mechanism for the persistent antifibrotic effect is that the infusion of macrophages recruits innate immune cells, such as monocytes and neutrophils, to the fibrotic liver to promote collagen degradation and improve liver function.^[^ ^6^, ^9^ ^]^ In our study, infusion of Mφ*^tgmif^* showed strong chemotactic potential, which may be attributed to the extremely high level of CCL2 expression. Hence, the long‐lasting therapeutic effect of Mφ*^tgmif^* could be caused by their chemotactic potential to modulate the hepatic immune microenvironment. Ly6C^hi^ pro‐inflammatory macrophages dominated the hepatic macrophage population in chronically inflamed and fibrotic mouse livers.^[^ ^33^ ^]^ When fiber resolution starts, these Ly6C^hi^ macrophages phenotypically switch to the Ly6C^lo^ subset, which represents the principle MMP‐expressing population during fibrosis resolution.^[^ ^17b^ ^]^ MMPs are critical regulatory components that mediate the degradation of the ECM, while TIMPs promote synthesis and inhibit the degradation of ECM by inhibiting MMPs.^[^ ^5^, ^34^ ^]^ Our data here revealed that the phenotype of these MoMFs recruited by Mφ*^tgmif^* was restorative Ly6C^lo^ macrophages. The Ly6C^lo^ macrophages in the Mφ*^tgmif^*‐treated group were significantly increased with the high regulation of CX3CL1, suggesting that CX3CL1 could promote Ly6C^hi^ pro‐inflammatory macrophages maturation into Ly6C^lo^ restorative macrophages.^[^ ^19^ ^]^ Infusion Mφ*^tgmif^* upregulated the MMP/TIMP ratio to degrade the extracellular matrix and reduce liver fibrosis.

Intriguingly, it was inappropriate for the r*Tg*MIF protein to ameliorate liver fibrosis (Figure 5c,d). Treatment with r*Tg*MIF directly activated HSCs with increased expression of α‐SMA and COL‐1, whereas Mφ*^tgmif^* deactivated HSCs. A recent study reported that *T. gondii* infection is characterized by the great induction of host chemokine expression, especially CCL2, rather than by the induction of IL‐12.^[^ ^35^ ^]^ The results suggested that some parasite‐derived molecules trigger host CCL2 production in a cell‐intrinsic manner. Consistent with this notion, our results revealed that the CCL2 expression level in Mφ*^tgmif^* was much higher (≈3691.5‐fold) than that in the control group. Mφ*^tgmif^* performed its function in vivo mainly through chemotaxis, modulating the hepatic immune microenvironment, with the overall effect of deactivating aHSCs and reducing liver collagen, which is in line with the idea that the recruitment of host innate immune cells to reinforce injury repair mechanisms, and it is a critical mechanism for macrophage therapy. Therefore, the immune microenvironment was regulated by the strong chemotactic potential in Mφ*^tgmif^*, but not r*Tg*MIF itself, to suppress the activation of HSCs.

It should be stressed here that we administered Mφ*^tgmif^* at a stage when liver fibrosis was already evident. This is more in line with the actual clinical situation. That is, when a patient is diagnosed with liver fibrosis, further treatment will be initiated. Although Mφ*^tgmif^* has some extent of low‐grade inflammation, its main function is to recruit monocyte‐derived macrophages and then facilitate the transition from fibrogenesis to fibrosis regression. Moreover, based on the fact that peripherally injected Mφ*^tgmif^* predominantly accumulated in the liver and survived for just ≈7 days to exert a therapeutic effect, Mφ*^tgmif^* could not cause long‐term effects. However, the current study has some limitations. First, we focused on MoMFs and HSCs, the main cell types that affect liver fibrosis. Testing of the effect of Mφ*^tgmif^* on other types of cells, including but not limited to endothelial cells, hepatocytes, or other immune cells, should be performed. Second, we need to further explore the mechanisms of BMDMs*^tgmif^* on liver fibrosis, such as attempting to neutralize CX3CL1 in vivo. Third, the study of *Tg*MIF‐transgenic human monocyte‐derived macrophages should be further examined to facilitate clinical translation. Finally, the Mφ*^tgmif^* still needs to assess its effectiveness against other types of liver fibrosis, such as nonalcoholic steatohepatitis (NASH) induced liver fibrosis.

In conclusion, *Tg*MIF‐transgenic macrophages with stable pro‐resolution and strong chemotactic capacity effectively ameliorated liver fibrosis by modulating the immune microenvironment and promoting fibrinolysis. Our findings do not merely suggest a viable immunotherapy for liver fibrosis but also confirm a therapeutic strategy based on the evolutionarily designed immunomodulator to treat human diseases.

## 4 Experimental Section

### Plasmid and Stable Cell Line Construction

The mRNA from the *Toxoplasma gondii* ME49 strain was isolated and reverse‐transcribed into cDNA. The *Tg*MIF gene was amplified using PCR. The primers were designed as follows: forward 5′‐CCGGAATTCGCCACCATGCCCAAGTGCATGATCTTTTGCC‐3′ (the EcoRI restriction site was marked with the underline) and reverse 5‘‐ATTTGCGGCCGCAGCCGAAAGTTCGGTCGCCCATGGCC‐3′ (the NotI restriction site was marked with the underline). Primer synthesis and gene sequencing were completed by Virotherapy Technologies (Wuhan, China). Recombinant lentivirus encoding *Tg*MIF was constructed using the pLVX‐3FLAG‐ZsGreen‐Puro vector or pLVX‐Myc‐Antares2‐Puro vector. The murine macrophage cell line RAW264.7 (Mφ) was purchased from Stem Cell Bank, the Chinese Academy of Sciences. Using a lentiviral vector (LV) system, we successfully established 4 stable cell lines. Mφ*^tgmif^* : Mφ stably expressing LV‐*Tg*MIF‐ZsGreen; Mφ^LV^: Mφ stably expressing LV‐ZsGreen; Mφ*^tgmif^‐*2: Mφ stably expressing LV‐*Tg*MIF‐Antares2; Mφ^LV^‐2: Mφ stably expressing LV‐Antares2.

### Isolation of Human Monocyte‐Derived Macrophages (hMDMS)

Healthy donors were enrolled in the First Affiliated Hospital of Anhui Medical University in Hefei City, Anhui Province, China. Approximately 4 mL of venous blood was drawn from each participant. hMDMS were prepared as described.^[^ ^36^ ^]^ The research protocol was approved by the ethics committee of the First Affiliated Hospital of Anhui Medical University (permit number: PJ2022‐14‐40). Written informed consent was provided by all study participants.

### Animals and Liver Fibrosis Model

Male 6‐ to 7‐week‐old C57BL/6 mice were purchased from the Experimental Animal Center of Anhui Medical University, housed with a 12 h light/dark cycle, and allowed free access to normal food and water. All mice were acclimatized for 1 week before experiments and maintained under specific pathogen‐free (SPF) conditions. Mice received 0.6 mL kg^−1^ body weight CCl_4_ (Sigma–Aldrich, St. Louis, MO, USA) diluted in olive oil by intraperitoneal (i.p.) injection twice per week to induce liver fibrosis. As a control, mice were injected with an equal volume of olive oil. The BDL and sham operation surgical procedures were performed under aseptic conditions. All experiments in mice were approved by the Animal Ethical Committee of Anhui Medical University (permit number: 20 180 145), and efforts were made to minimize the number of animals used and the animals suffering pain during the experimental process.

### Isolation and Culture of Bone Marrow‐Derived Macrophages (BMDMs)

In brief, C57BL/6 mice aged 4–5 weeks were sacrificed humanely. The hind legs and the excess fascia of the mouse were removed. All the cells in the marrow cavity were blown out with cold PBS containing 2% FBS. The cells were washed twice with cold PBS and then incubated in complete RPMI 1640 medium for 5 h at 37 °C with 5% CO_2_ to remove stromal cells. The media was carefully collected in 15 mL Falcon tubes after 5 h. The samples were centrifuged (1,400 rpm) at 4 °C for 5 min to pellet the cells. Then, the BM cells were counted and resuspended in complete RPMI 1640 at a density of 2 × 10^6^ cells mL^−1^ Place 1 mL of cells into a 10 cm dish and then add the lentivirus to the dish at an MOI of 20. The cells were incubated at 37 °C and 5% CO_2_ for 48 h. Then, the medium containing lentivirus was carefully aspirated and replaced with a differentiation medium (complete RPMI 1640 medium containing 30% L929 cell culture medium). The cells were cultured for an additional 7 days, and the medium was changed to a fresh medium every 2 days. BMDMs were stimulated for 24 h with PBS, lipopolysaccharide (LPS, 50 ng mL^−1^, Sigma), and IFN‐γ (20 ng mL^−1^, Peprotech).

### RNA Extraction and Quantitative Real‐Time Polymerase Chain Reaction (qRT–PCR)

Total RNA of cultured cells or liver tissue was extracted using TRIzol reagent (Invitrogen Life Technologies, Carlsbad, CA, USA) and transcribed into cDNA by using Evo M‐MLV RT Premix for qPCR (AG, Changsha, Hunan, China) following the manufacturer's instructions. qRT–PCR was performed using the SYBR Green Premix Pro Taq HS qPCR Kit (AG, Changsha, Hunan, China) on a Roche LightCycler 96 (Roches, Basel, Switzerland), with β‐actin as an internal control. All experiments were performed in triplicate (cultured cells) or at least sextuplicate (liver tissue).

### RNA‐seq

Ensemble genes were used as gene annotations. Reads with low quality and adapters were trimmed by Trimmomatic v0.39 and then aligned by STAR (version 2.7.2b) with default parameters. Then, filtering was performed to remove alignments with MAPQ < 50. The unique reads were used to calculate the fragments per kilobase of exon per million fragments mapped (FPKM) with Cufflinks (version 2.2.1) (http://cufflinks.cbcb.umd.edu). Genes were filtered with FPKM < 1 in all cell types. To identify differentially expressed genes between Mφ^LV^ and Mφ*^tgmif^* or between LPS/IFNγ‐Mφ and Mφ*^tgmif^*, HOMER software command getDiffExpression.pl with –DESeq2 was employed. FDR <0.001 and log2 |fold change| > 1 were used as cutoffs for significantly differentially expressed genes. Differentially expressed genes were analyzed using Metascape (http://metascape.org) for functional enrichment.

### Bioluminescence Imaging

RAW264.7‐cell lines stably expressing luciferase Antares2 (using the pLVX‐*Tg*MIF‐Myc‐Antares2‐Puro plasmid) were generated and subjected to a bioluminescence imaging assay. A total of 2 × 10^6^ luciferase‐expressing macrophages were injected into C57BL/6 mice through the tail vein. Noninvasive bioluminescence imaging was performed at 3 h, day 1, day 3, day 7, and day 14 following cell infusion. Five minutes before imaging, each mouse received an intravenous injection of 0.3 µmol diphenylterazine (MCE, HY‐111382, China) formulated with a mixture of organic cosolvents. Images were captured by an optical imaging system (Spectral Instruments Imaging, Tucson, AZ, USA).

### Serum Biochemical Analysis

Serum levels of alanine aminotransferase (ALT) and aspartate aminotransferase (AST) were determined by an automatic biochemical analyzer (Automatic Analyzer 3100, Hitachi, Tokyo, Japan).

### Isolation of Hepatic Macrophages and Hepatic Stellate Cells (HSCs)

Livers were perfused in situ with warmed (37 °C) HBSS solution, followed by incubation with collagenase type IV buffer (Sigma–Aldrich, St. Louis, MO, USA). The perfused livers were minced with scissors and filtered through 70 µm nylon mesh cell strainers. Non‐parenchymal cells (NPCs) were separated from hepatocytes by low‐speed centrifugation (50×g, 5 min) at 4 °C. Hepatic macrophages were isolated by a two‐step 50%/25% Percoll (GE Healthcare, USA) gradient and centrifuged at 1800×g at 4 °C for 15 min. The cells between the layers of the 25 and 50% Percoll solutions were carefully extracted for further assays. For HSCs isolation, NPCs were separated by centrifugation at 1400×g on a Nycodenz density gradient for 20 min at 4 °C without a brake, following a previously established protocol.^[^ ^37^ ^]^ Isolated HSCs were cultured in DMEM containing 10% fetal bovine serum (FBS; Gibco, Grand Island, NY, USA) and 1% P/S in a humidified atmosphere of 5% CO_2_ at 37 °C.

### Flow Cytometry Assay

The cell suspension was prepared, and the cell density was adjusted to 2 × 10^7^ cells mL^−1^ after cell counting. A total of 100 µL of cell suspension was taken and incubated with 5% rat serum to block the nonspecific binding of the primary antibody. Then, the cells were stained with a combination of fluorochrome‐conjugated antibodies, incubated on ice for 30 min, and washed twice with PBS. Samples were measured on a CytoFlex System (Beckman Coulter, Brea, CA, USA). Data were analyzed using CytExpert software v2.4.

### Statistical Analysis

Statistical analyses were performed using the SPSS software (SPSS version 26; IBM SPSS). Data were expressed as the mean ± SD. A two‐tailed Student's t‐test was applied to compare two groups and a one‐way ANOVA analysis was applied for the comparison of multiple groups. Post hoc analysis was conducted using the Least Significant Difference (LSD) method. *P* values <0.05 were considered statistically significant.

## Conflict of interest

The authors declare no conflict of interests.

## Author Contributions

Y.C., J.W., and N.Z. contributed equally to this work. Y.C., J.W., and N.Z. performed the experiments. J.D. and L.‐J. C. conceived the study. R.A. and H.‐J. C. were involved in the design of experiments. Y.C., J.W., N.Z., Q.F., Z.‐R. D., D.L., F.‐M. L. and Q.Y. analyzed data. X.‐P. C. and X.‐X. W. performed RNA‐seq data analysis. Y.C. wrote the manuscript. J.D. and L.J.C. did the critical revision of the article. All authors read and approved the submitted version.

## Supporting information

Supporting Information

## Data Availability

The data that support the findings of this study are available from the corresponding author upon reasonable request.
