# Supplementary material for: Protozoan‐Derived Cytokine‐Transgenic Macrophages Reverse Hepatic Fibrosis
Source: Adv Sci (Weinh). 2024 Jan 21;11(13):2308750. doi: 10.1002/advs.202308750 (PMC10987136; doi:10.1002/advs.202308750)
Supplement: Supplementary file 1 — Supporting Information [file ADVS-11-2308750-s001.pdf]

ADVANCED SCIENCE
Open Access

Supporting Information

for *Adv. Sci.*, DOI 10.1002/advs.202308750

Protozoan-Derived Cytokine-Transgenic Macrophages Reverse Hepatic Fibrosis

Ying Chen, Jie Wang, Nan Zhou, Qi Fang, Haijian Cai, Zhuoran Du, Ran An, Deng Liu, Xuepeng Chen, Xinxin Wang, Fangmin Li, Qi Yan, Lijian Chen and Jian Du**

Supporting Information for

Protozoan-derived Cytokine-Transgenic Macrophages Reverse Hepatic Fibrosis

Ying Chen[1, 2, 4, #], Jie Wang[1, 2, #], Nan Zhou[1, 2, #], Qi Fang[3], Haijian Cai[1, 2], Zhuoran Du[6], Ran An[1, 2], Deng Liu[3], Xuepeng Chen[5], Xinxin Wang[5], Fangmin Li[1, 2], Qi Yan[1, 2], Lijian Chen[3, *], Jian Du[1, 2, *]

[1] *Department of Biochemistry and Molecular Biology, Research Center for Infectious Diseases, School of Basic Medical Sciences, Anhui Medical University, Hefei 230032, China*

[2] *The Provincial Key Laboratory of Zoonoses of High Institutions in Anhui, Anhui Medical University, Hefei 230032, China*

[3] *Department of Anesthesiology, The First Affiliated Hospital of Anhui Medical University, Hefei 230032, China*

[4] *School of Nursing, Anhui Medical University, Hefei 230032, China*

[5] *GMU-GIBH Joint School of Life Sciences, The Guangdong-Hong Kong-Macau Joint Laboratory for Cell Fate Regulation and Diseases, Guangzhou National Laboratory, Guangzhou Medical University, Guangzhou 510005, China*

[6] *Department of Clinical Medicine, Wannan Medical College, Wuhu 241002, China*

[#] *These authors contributed equally to this work.*

Address for Correspondence:

*To whom correspondence may be addressed. Email: *chenlijian77@126.com, or dujian@ahmu.edu.cn*

This PDF file includes:

Table S1 for supplementary table

Fig S1 to S10 for multiple supplementary figures

Table S1. Primers for qRT–PCR analysis (mouse).

Target	Forward primer（5’-3’）	Reverse primer(5’-3’)
*Tg*MIF	ATGCCCAAGTGCATGATCTTTTG	GCCGAAAGTTCGGTCGCC
CCL-2	CAGGTCCCTGTCATGCTTCT	GTCAGCACAGACCTCTCTCT
IL-1β	TCCAGGATGAGGACATGAGCAC	GAACGTCACACACCAGCAGGTTA
iNOS	GCAGAGATTGGAGGCCTTGTG	GGGTTGTTGCTGAACTTCCAGTC
TIMP-1	CGAGACCACCTTATACCAGCG	ATGACTGGGGTGTAGGCGTA
MMP-9	CCATGCACTGGGCTTAGATCA	GGCCTTGGGTCAGGCTTAGA
CX3CL-1	ACGAAATGCGAAATCATGTGC	CTGTGTCGTCTCCAGGACAA
COL-1	GCTCCTCTTAGGGGCCACT	ATTGGGGACCCTTAGGCCAT
TGF-β	GGATACCAACTATTGCTTCAGCTCC	AGGCTCCAAATATAGGGGCAGGGTC
α-SMA	CCCAGACATCAGGGAGTAATGG	TCTATCGGATACTTCAGCGTCA

Figure S1

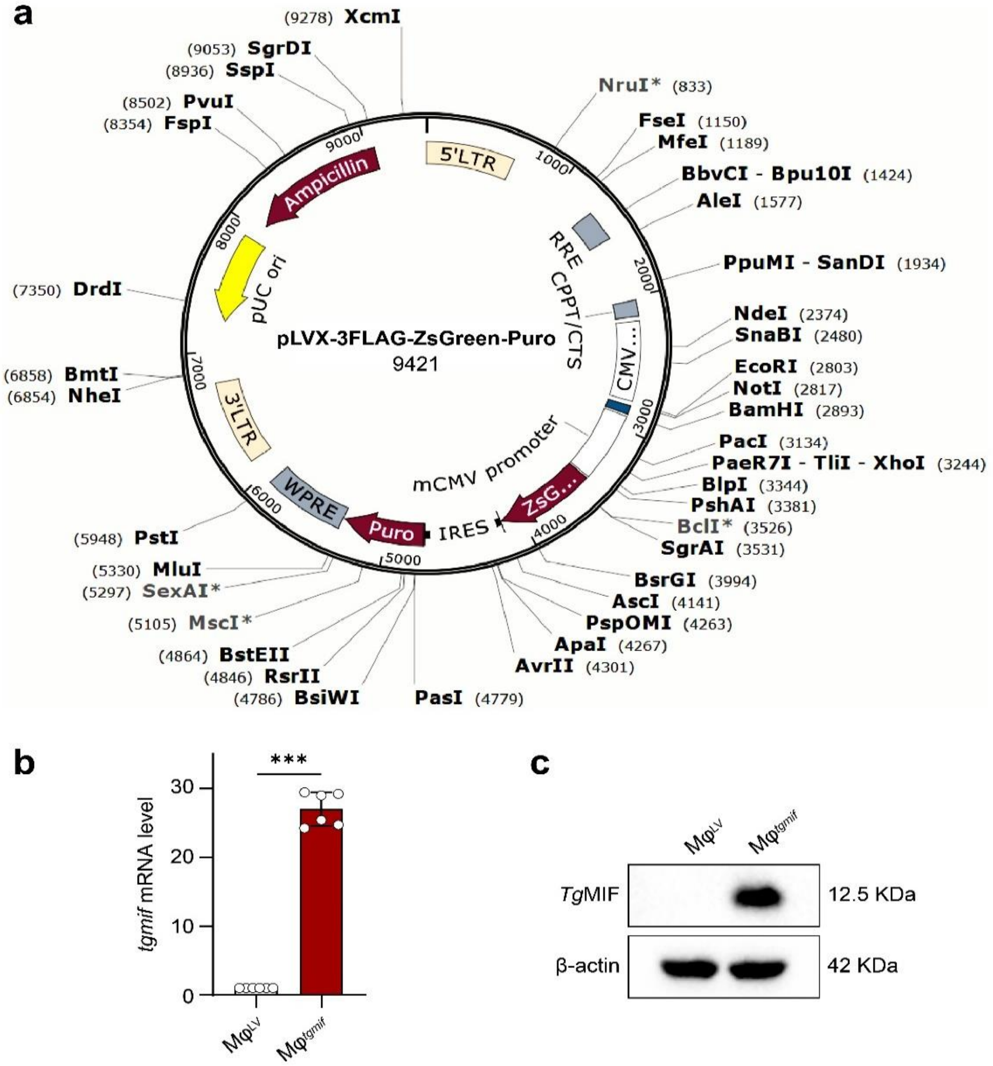

Figure. S1. The expression of *Tg*MIF in stable cell lines.

a. Map of the pLVX-3FLAG-ZsGreen-Puro vector. **b.** The expression of *tg*mif gene in two stable cell lines $M\varphi^{tgmif}$ and $M\varphi^{LV}$ were examined through qRT–PCR (n=6 per group). Results were analyzed using a two-tailed t test. Bars=mean±SD. ***P < 0.001. **c.** The expression level of *Tg*MIF protein in stable cell lines were examined by Western blot (WB) analysis.

Figure S2

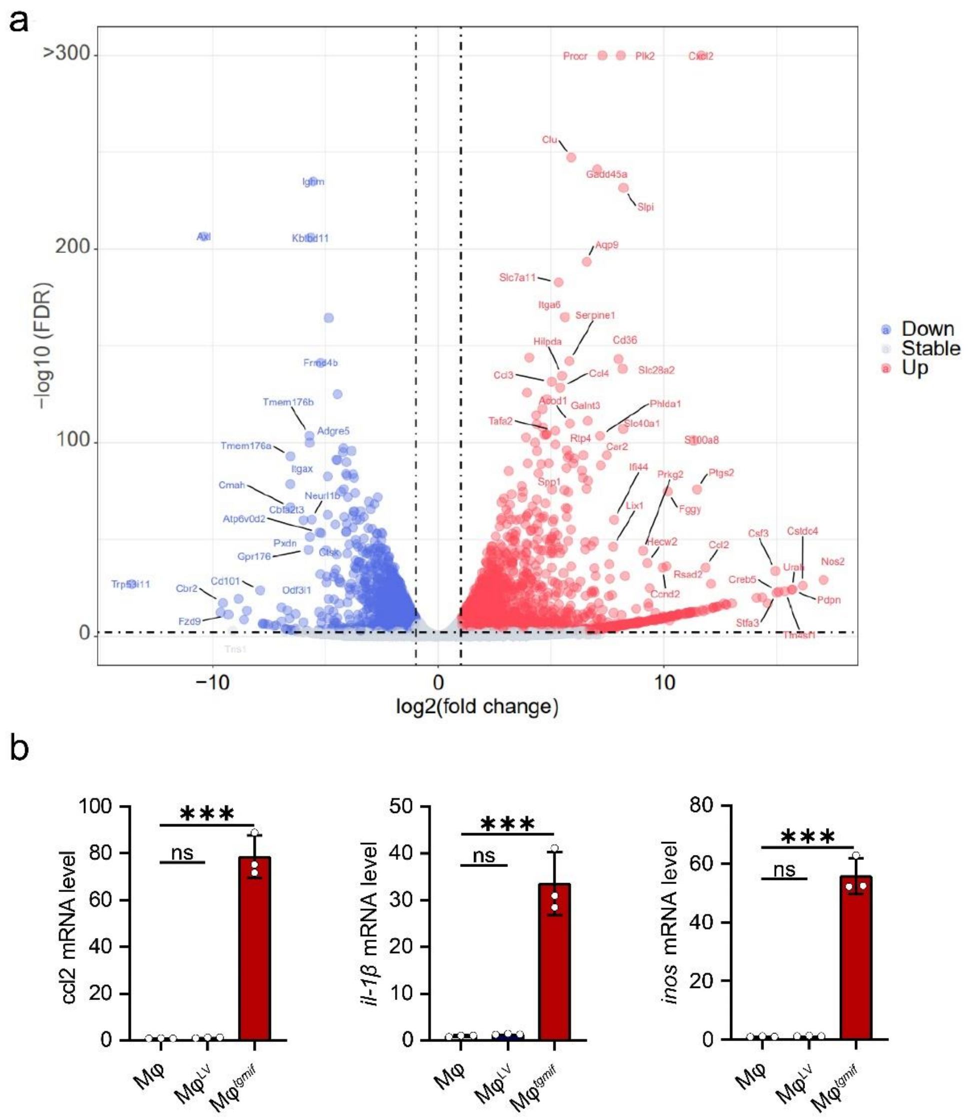

Figure. S2. Volcano plot of gene expression profiles in Mφ^{tgmif} and Mφ^{LV} samples. **a.** Volcano plot showed the differentially expressed genes between Mφ^{tgmif} and Mφ^{LV}, with FDR < 0.001 and absolute log fold change (FC) > 1; the red dots represent upregulated genes, the blue dots represent downregulated genes, and the gray dots represent genes with similar expression levels between the groups. **b.** The RNA-seq data was verified using qPCR. Quantification was shown (n=3 per group). The results were analyzed using one-way ANOVA. Bars = mean ± SD. ***P < 0.001.

Figure S3

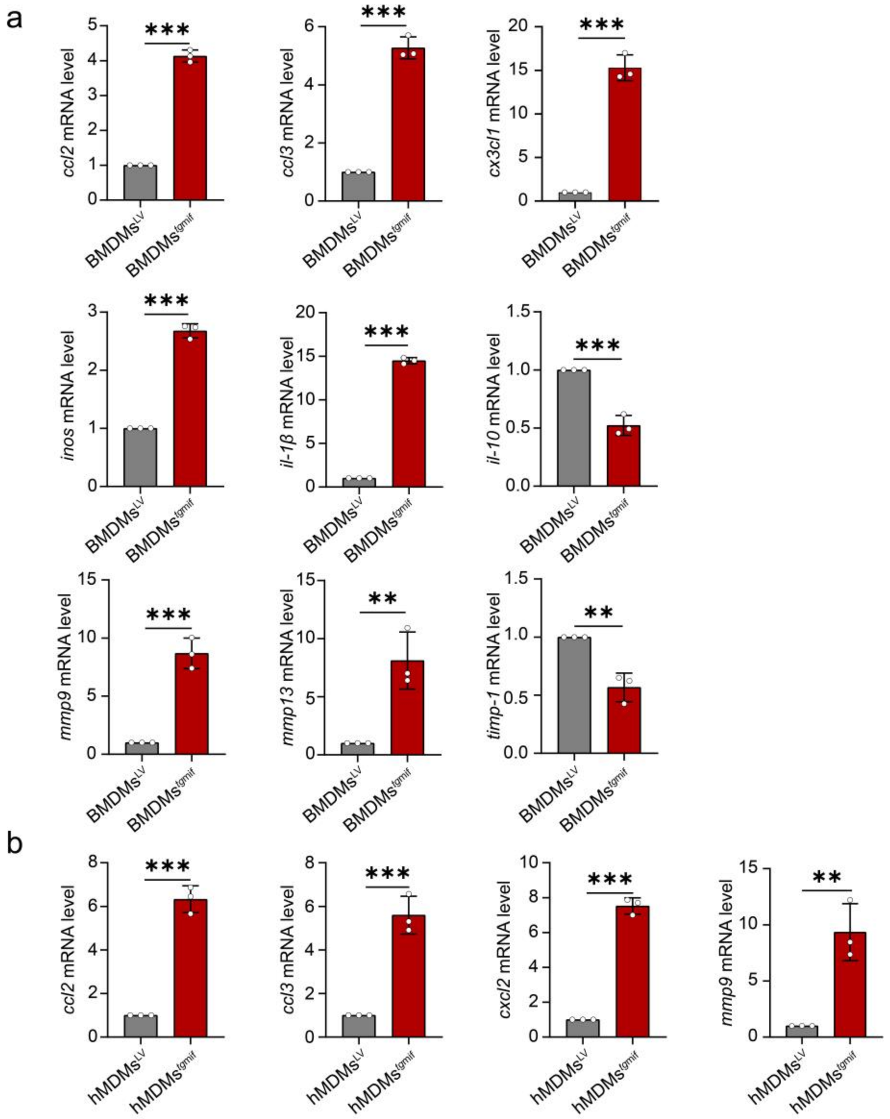

Figure. S3. Verify the key screened results on BMDMs and hMDMs.

a. Bone marrow-derived macrophages (BMDMs) were extracted and subjected to lentiviral transduction for overexpression of *Tg*MIF. BMDMs^{tgmif}: BMDMs expressing LV-*Tg*MIF-ZsGreen; BMDMs^{LV}: BMDMs expressing LV-ZsGreen. Gene expressions were determined by qRT-PCR (n=3 per group). **b.** Human monocyte-derived macrophages (hMDMs) were extracted and subjected to lentiviral transduction for overexpression of *Tg*MIF. hMDMs^{tgmif}: hMDMs expressing LV-*Tg*MIF-ZsGreen; hMDMs^{LV}: hMDMs expressing LV-ZsGreen. Gene expressions were determined by qRT-PCR (n=3 per group). The results were analyzed using a two-tailed Student's t test. Bars = mean ± SD. $^{**}P < 0.005$, $^{***}P < 0.001$.

Figure S4

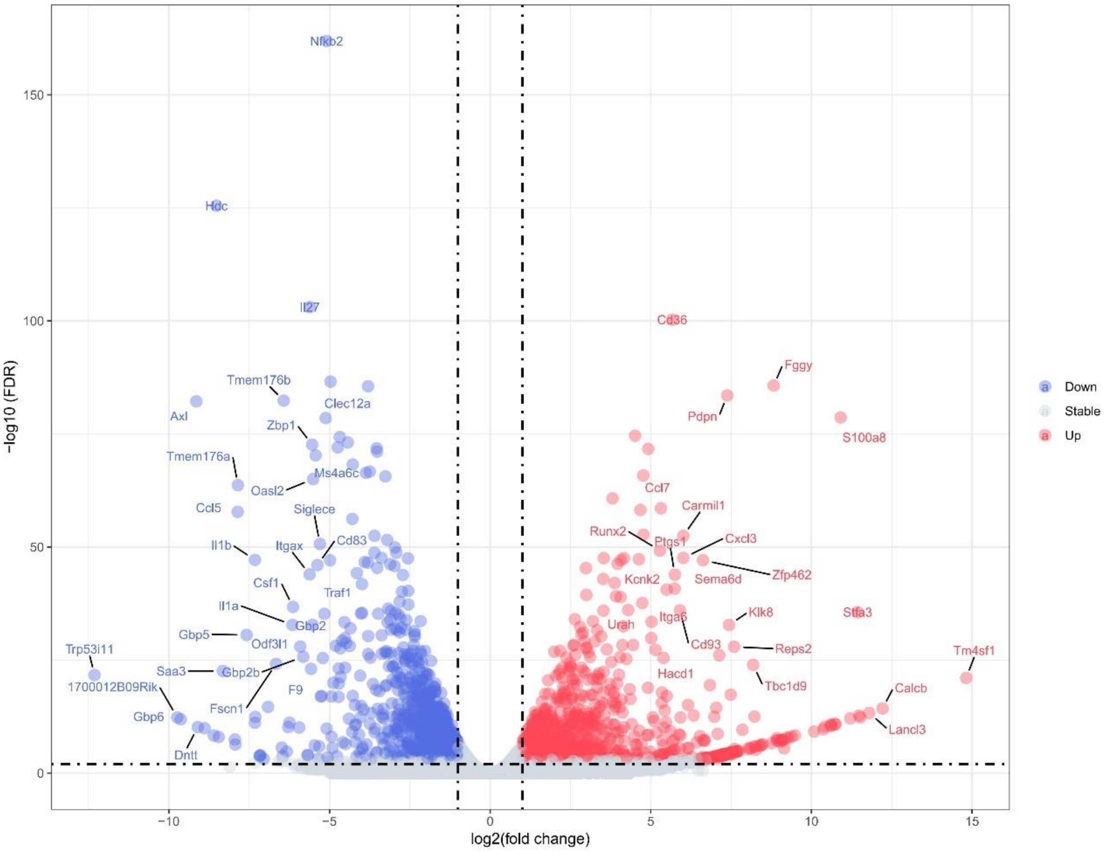

Figure. S4. Volcano plot of the gene expression profile in Mφ*tgmif* and LPS/IFN-γ-Mφ samples.

Volcano plot showing the differentially expressed genes between Mφ*tgmif* and LPS/IFN-γ-Mφ, with FDR < 0.001 and absolute log fold change (FC) > 1; the red dots represent upregulated genes, the blue dots represent downregulated genes, and the gray dots represent genes with similar expression levels between the groups.

Figure S5

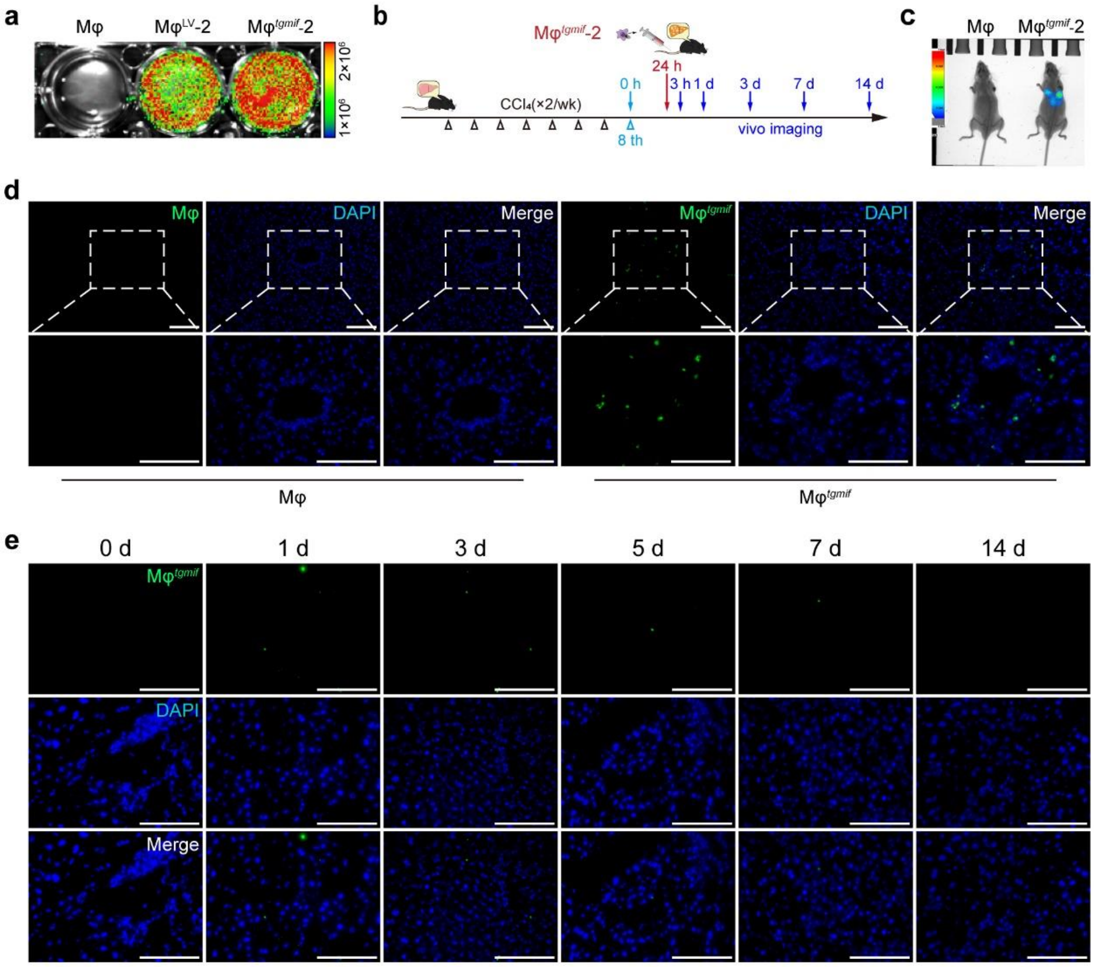

Figure. S5. Infused macrophages engraft in liver tissues.

a. Bioluminescence showed that Antares2-expressing macrophages had strong bioluminescence signals *in vitro*. **b.** Study design: Mice were infused with macrophages (2×10^6/mouse) one day after the 8th CCl_4 injection through the tail vein and examined at 3 h, 1 d, 3 d, 7 d and 14 d. **c.** Whole-body imaging using IVIS at 10 min after infusion. Mφ: RAW264.7; $M\varphi^{LV}$-2: Mφ stable expression of LV-Antares2; $M\varphi^{tgmif}$-2: Mφ stable expression of LV-*Tg*MIF-Antares2. **d.** To further confirm that macrophages indeed reached the liver, we infused CCl_4-induced fibrotic mice with ZsGreen-labeled macrophages. Mice were sacrificed humanely 24 hours after injection. The liver tissues were collected, and frozen sections were prepared and visualized under a fluorescence microscope (above pictures display magnification: ×200, below pictures display magnification: ×400, Scale bar=100 μm). **e.** Immunofluorescent staining of the fresh-frozen sections as indicated time points (Scale bar=100 μm).

Figure S6

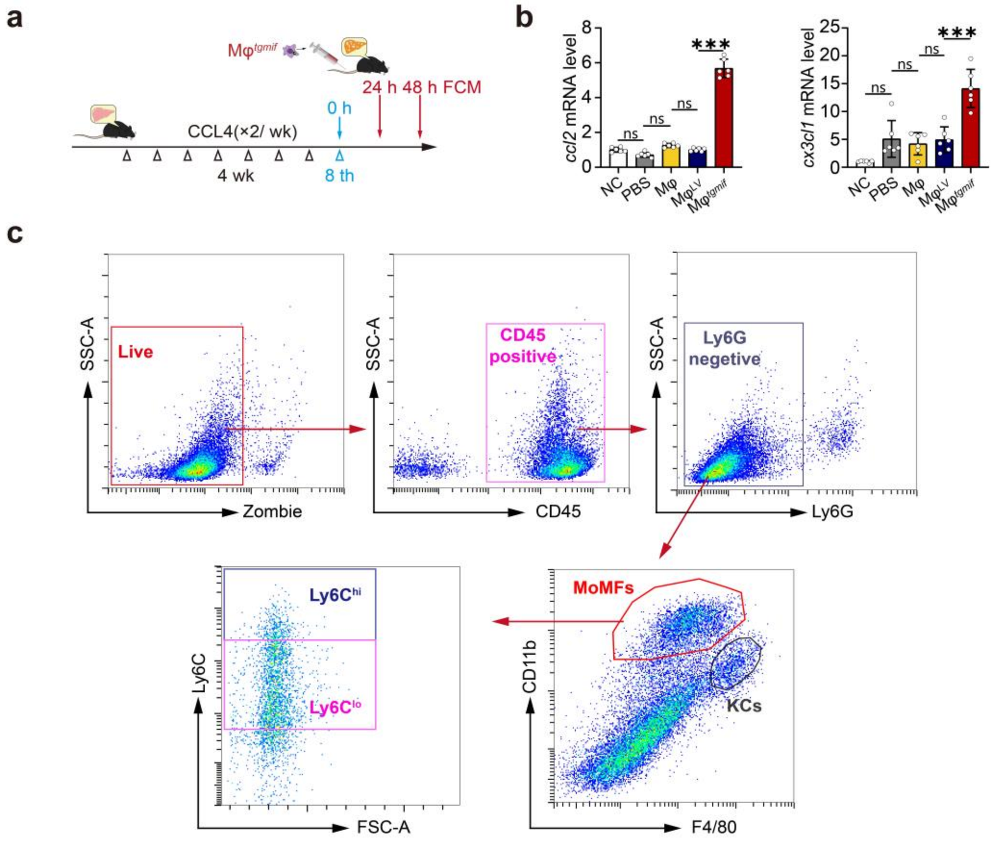

Figure. S6. Characterization of MoMFs and KCs.

a. Study design: Mice were infused with 2×10^6 macrophages (diluted in 150 µl PBS) or 150 µl PBS through the tail vein at 24 h post-8th CCl_4 injection. Mice were euthanized 1 day after injection. **b.** The relative mRNA expression levels of cx3cl1 and ccl2 in liver tissue were determined by qRT–PCR (n=6 per group). **c.** Hepatic macrophages were isolated 24 hours after cell infusion and analyzed by flow cytometry. Cells were gated to identify MoMFs ($CD45^+Ly6G^-CD11b^{hi}F4/80^{int}$) and KCs ($CD45^+Ly6G^-CD11b^{lo}$ $F4/80^{hi}$). These MoMFs were further divided into proinflammatory MoMFs ($Ly6C^{hi}$) and restorative MoMFs ($Ly6C^{lo}$).

Figure S7

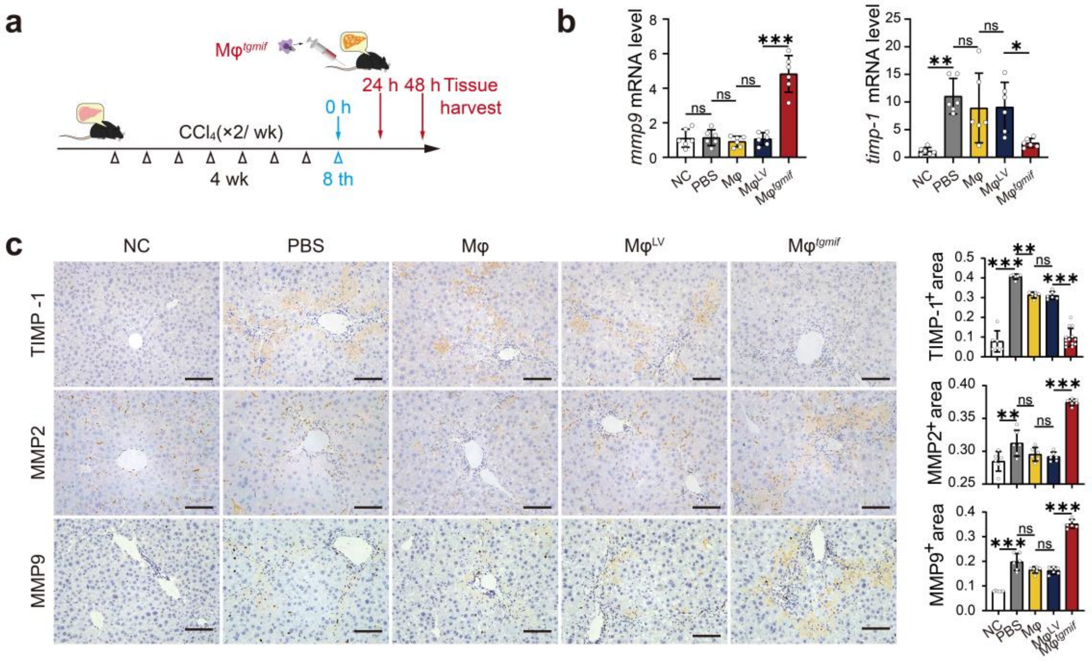

Figure. S7. $M\varphi^{tgmif}$ regulates the MMP/TIMP ratio.

a. Study design: Mice were infused with 2×10^6 macrophages (diluted in 150 µl PBS) or 150 µl PBS through the tail vein at 24 h post-8th CCl_4 injection. Mice were euthanized 24 hours after inoculation. **b.** The relative mRNA expression levels of mmp9 and timp-1 in liver tissue were determined by qRT–PCR (n=6 per group). **c.** The expression of TIMP-1, MMP2 and MMP9 in livers tested by IHC (×200; Scale bar=200 µm). Positively stained regions were quantitatively analyzed on the right (n = 6/6/6/6/13 (TIMP-1) per group; n=6 per group (mmp2 and mmp 9)). Results were analyzed using one-way ANOVA. Bars = mean ± SD. *P < 0.05, **P < 0.01, ***P < 0.001 and ns, not statistically significant. NC: negative control, mice were treated with an equal amount of pure olive oil; PBS: fibrotic mice received PBS; Mφ: fibrotic mice received RAW264.7; $M\varphi^{LV}$: fibrotic mice received $M\varphi^{LV}$ stable cell line; $M\varphi^{tgmif}$: fibrotic mice received $M\varphi^{tgmif}$ stable cell line.

Figure S8

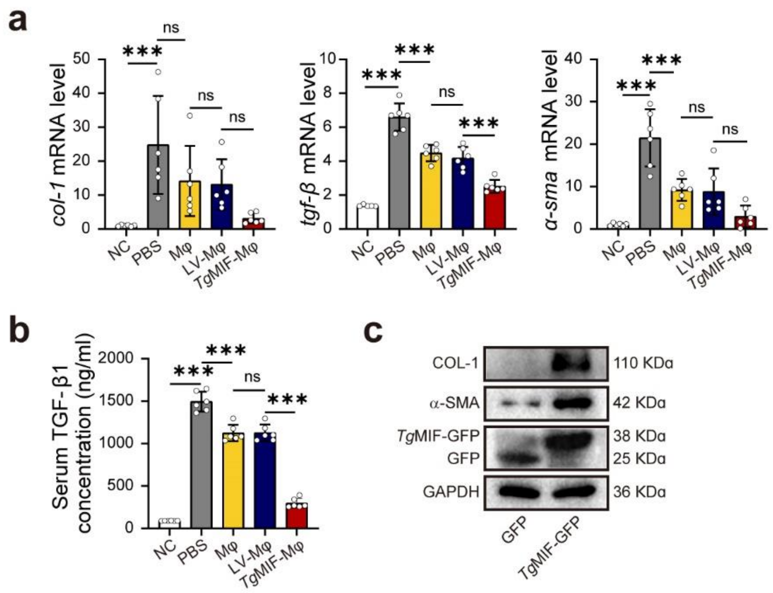

Figure. S8. Mφ*tgmif* but not *Tg*MIF deactivates HSCs.

a. The relative mRNA expression levels of col-1, tgf-β, and α-SMA in liver tissue were determined by qRT–PCR (n=6 per group). **b.** ELISA was used to detect the release levels of TGF-β1 in the serum of mice (n=6 per group). The results were analyzed using one-way ANOVA. Bars = mean ± SD. ***P < 0.001 and ns, not statistically significant. NC: negative control, mice were treated with an equal amount of pure olive oil; PBS: fibrotic mice receive PBS; Mφ: fibrotic mice received RAW264.7; $Mφ^{LV}$: fibrotic mice received $Mφ^{LV}$ stable cell line; $Mφ^{tgmif}$: fibrotic mice received $Mφ^{tgmif}$ stable cell line. **c.** Protein expression in LX-2 cells transfected with *Tg*MIF-GFP or GFP (control) was detected by WB.

Figure S9

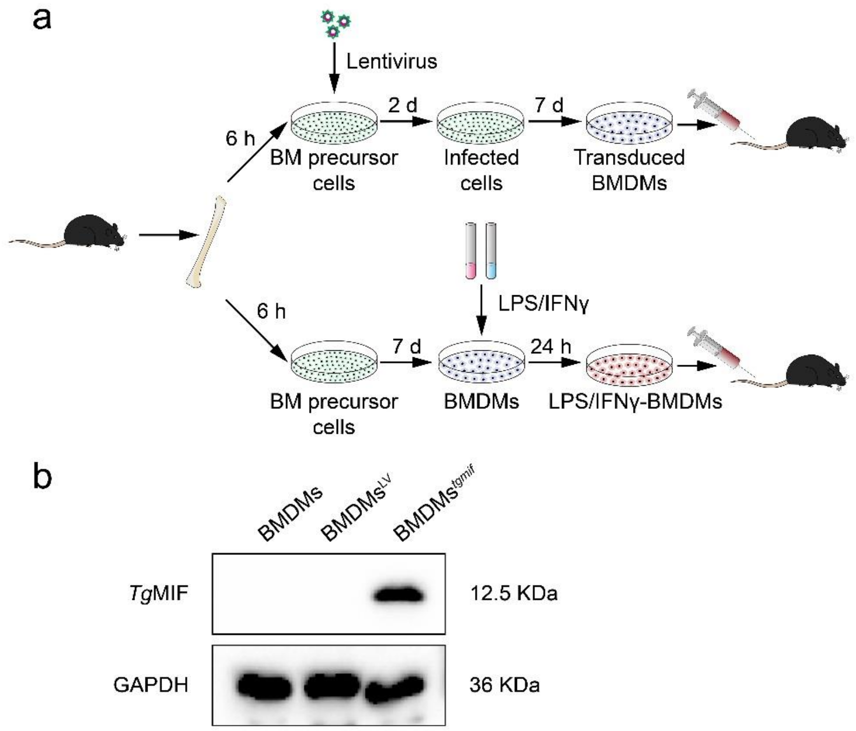

Figure. S9. The expression of *Tg*MIF in BMDMs^{tgmif}.

a. BMDMs lentiviral transduction and LPS/IFN-γ-BMDMs construction. **b.** The expression of *Tg*MIF in different groups of BMDMs. GAPDH was detected as the loading control.

Figure S10

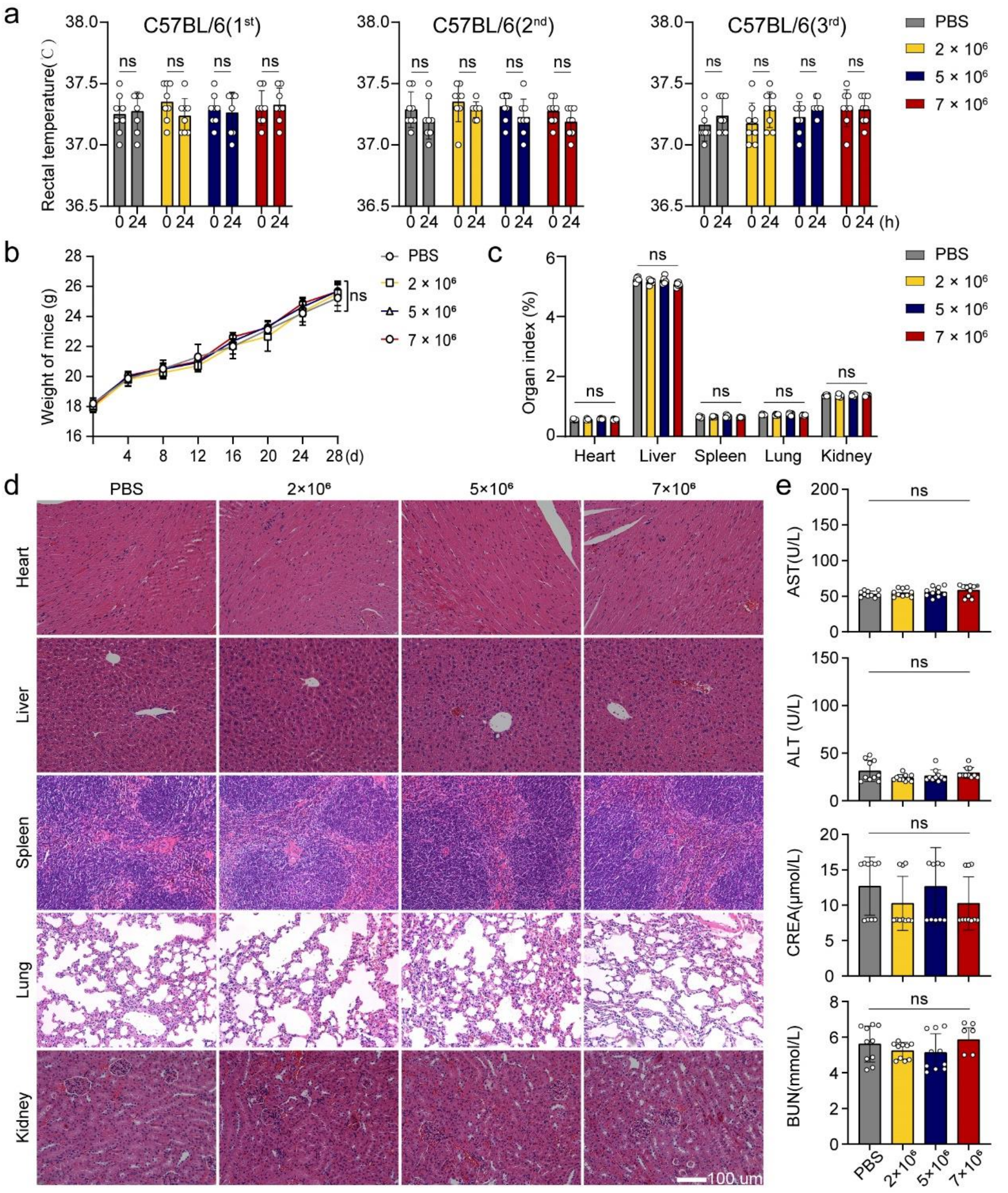

Figure. S10. Assess the systemic safety and toxicity of BMDMs^{tgmif}.

a. C57BL/6 mice were given three dosages of BMDMs (2×10^6, 5×10^6, and 7×10^6 diluted in 150 μl PBS, respectively) or 150 μl PBS via the tail vein weekly for three weeks, and rectal temperature was monitored immediately before and 24 hours after each injection (n=8 per group). **b.** C57BL/6 mice were administered three dosages of BMDMs^{tgmif} weekly for three weeks. Body weight of the mice was monitored prior to injection and every 4 days until 4 weeks (n=6 per group). **c-e.** C57BL/6 mice were administered three dosages of BMDMs^{tgmif} weekly for three weeks. After the third injection, the mice were

sacrificed humanely one week later and the organ index (n=6 per group) **(c)**, the damage status of the major organs **(d)** as well as liver and kidney function (n=10 per group) **(e)**, were evaluated. The results were analyzed using one-way ANOVA. Bars = mean ± SD. ns, not statistically significant.
